# Optimistic vs Pessimistic Message Framing in Communicating Prognosis to Parents of Very Preterm Infants: The COPE Randomized Clinical Trial

**DOI:** 10.1001/jamanetworkopen.2024.0105

**Published:** 2024-02-23

**Authors:** Fiona A. Forth, Florian Hammerle, Jochem König, Michael S. Urschitz, Philipp Neuweiler, Eva Mildenberger, André Kidszun

**Affiliations:** 1 Division of Neonatology, Center for Pediatric and Adolescent Medicine, University Medical Center of the Johannes Gutenberg-University Mainz, Mainz, Germany; 2 Department of Pediatric and Adolescent Psychiatry and Psychotherapy, University Medical Center of the Johannes Gutenberg-University Mainz, Mainz, Germany; 3 Division of Pediatric Epidemiology, Institute for Medical Biostatistics, Epidemiology and Informatics, University Medical Center of the Johannes Gutenberg-University Mainz, Mainz, Germany; 4 Journalistisches Seminar, Johannes Gutenberg-University Mainz, Mainz, Germany; 5 Division of Neonatology, Department of Pediatrics, Inselspital, Bern University Hospital, University of Bern, Bern, Switzerland

## Abstract

**Importance:**

In the neonatal intensive care unit, there is a lack of understanding about how best to communicate the prognosis of a serious complication to parents.

**Objective:**

To examine parental preferences and the effects of optimistic vs pessimistic message framing when providing prognostic information about a serious complication.

**Design, Setting, and Participants:**

This crossover randomized clinical trial was conducted at a single German university medical center between June and October 2021. Eligible participants were parents of surviving preterm infants with a birth weight under 1500 g. Data were analyzed between October 2021 and August 2022.

**Interventions:**

Alternating exposure to 2 scripted video vignettes showing a standardized conversation between a neonatologist and parents, portrayed by professional actors, about the prognosis of a hypothetical very preterm infant with severe intraventricular hemorrhage. The video vignettes differed in the framing of identical numerical outcome estimates as either probability of survival and probability of nonimpairment (optimistic framing) or a risk of death and impaired survival (pessimistic framing).

**Main Outcomes and Measures:**

The primary outcome was preference odds (ratio of preference for optimistic vs pessimistic framing). Secondary outcomes included state anxiety, perceptions of communication, and recall of numerical estimates.

**Results:**

Of 220 enrolled parents (142 [64.5%] mothers; mean [SD] age: mothers, 39.1 [5.6] years; fathers, 42.7 [6.9] years), 196 (89.1%) preferred optimistic and 24 (10.1%) preferred pessimistic framing (preference odds, 11.0; 95% CI, 6.28-19.10; *P* < .001). Preference for optimistic framing was more pronounced when presented second than when presented first (preference odds, 5.41; 95% CI, 1.77-16.48; *P* = .003). State anxiety scores were similar in both groups at baseline (mean difference, −0.34; −1.18 to 0.49; *P* = .42) and increased equally after the first video (mean difference, −0.55; 95% CI, −1.79 to 0.69; *P* = .39). After the second video, state anxiety scores decreased when optimistic framing followed pessimistic framing but remained unchanged when pessimistic framing followed optimistic framing (mean difference, 2.15; 95% CI, 0.91 to 3.39; *P* < .001). With optimistic framing, participants recalled numerical estimates more accurately for survival (odds ratio, 4.00; 95% CI, 1.64-9.79; *P* = .002) but not for impairment (odds ratio, 1.50; 95% CI, 0.85-2.63; *P* = .16).

**Conclusions and Relevance:**

When given prognostic information about a serious complication, parents of very preterm infants may prefer optimistic framing. Optimistic framing may lead to more realistic expectations for survival, but not for impairment.

**Trial Registration:**

German Clinical Trials Register (DRKS): DRKS00024466

## Key Points

**Question:**

Do parents of very preterm infants prefer optimistic or pessimistic message framing when informed of a serious complication in their child’s condition?

**Findings:**

This crossover randomized clinical trial using 2 scripted video vignettes depicting 2 types of message framing found that a clear majority of parents (89.1%) preferred the optimistic framing, while 10.9% preferred the pessimistic framing.

**Meaning:**

These results suggest that, when given prognostic information about a serious complication in their child’s condition, parents of very preterm infants may prefer optimistic framing.

## Teaser

This randomized clinical trial investigates whether parents of very preterm infants prefer optimistic or pessimistic framing in neonatologist communication of prognosis for a serious complication.

## Introduction

In the neonatal intensive care unit (NICU), very preterm infants represent a particularly vulnerable patient population. They are highly susceptible to postnatal complications such as intraventricular hemorrhage (IVH), which increases their risk of mortality and is a major cause of morbidity.^1,2,3^ Despite improved diagnostic capabilities and the increasing availability of long-term data on the outcome of very preterm infants, it remains a complex task for neonatologists to derive predictions for the short-term survival and long-term neurodevelopmental outcome of an individual infant from the results of general population-based research and to communicate these to parents.^4,5,6,7,8,9,10^ Moreover, physicians and parents are known to have different perspectives on the importance, discussion, and understanding of outcomes.^7,11^ Parents want and need prognostic information and communication tailored to their preferences.^4,11,12,13,14^ These are a prerequisite for developing realistic expectations for their child, adjusting to their role as parents, and participating in shared decision-making (SDM) as surrogates for their child.^8,15,16,17^ When communicating with parents, contextualizing the information to be conveyed can have a tremendous impact on their understanding.^18^

Although a number of studies have been conducted on the influence of different communication behaviors and message formulation, there is still insufficient knowledge about how parents of very preterm infants want to receive prognoses.^17,19,20,21,22,23^ It remains largely unclear how prognostic information should ideally be framed to meet parents’ preferences and what effects different framings of prognostic information may have in the NICU setting. The aim of this study was to examine parents’ preferences for optimistic vs pessimistic message framing and how such framing possibly affects emotional and cognitive outcomes.

## Methods

This randomized clinical trial (RCT) was approved by the ethics committee of the Medical Association of Rhineland-Palatinate. All participants provided electronic informed consent. The full trial protocol^24^ is available in Supplement 2. This report follows the Consolidated Standards of Reporting Trials (CONSORT) reporting guideline for RCTs.

### Trial Design, Setting, and Interventions

The COPE-Trial (Communicating prognosis to parents in the neonatal ICU: optimistic vs pessimistic) was a single-center randomized-controlled crossover trial, conducted at the Division of Neonatology of the University Medical Center Mainz (UMC Mainz) in Mainz, Germany. An experimental video vignette design^25,26,27^ was used with 2 video vignettes, portrayed by professional actors, depicting a conversation between a neonatologist and the parents of a hypothetical very preterm infant. The content of the conversation was the diagnosis of acute severe intraventricular hemorrhage in the infant and the associated prognosis. Many aspects of the 2 videos were standardized, including the setting, actors, flow of conversation, camera work, and duration. The message in both videos was logically equivalent but differed in presentation. Statistical outcome estimates for survival (50%) and impairment (50% in case of survival) were framed as either a probability of survival and probability of nonimpairment (optimistic framing) or a risk of death and impaired survival (pessimistic framing). In both videos, the nonverbal appearance of the neonatologist was congruent with the respective framing of the message. Message framing is interpreted as a broad concept in which the presentation of statistically identical information is modulated in a variety of ways.^28^ The scripts and the videos vignettes are provided as eMethods in Supplement 1.

### Participants and Procedures

Parents of surviving preterm infants with a birth weight under 1500 g treated at the UMC Mainz between January 2010 and December 2019 were eligible (906 in total) and included if they had sufficient German language skills (self-assessment). Individuals were excluded if they reported acute mental illness or persistent distress from the prematurity experience (self-report). Participants provided electronic informed consent prior to enrollment.

Participants were randomized to alternate exposure to 2 video sequences. Those randomized to the optimistic first group viewed the optimistic framing first, then the pessimistic framing, and vice versa in the pessimistic first group. Randomization was performed using computer-generated lists in blocks of variable length, stratified by participation of only the mother, only the father, and both parents. If both parents participated, they received the same allocation. Participants were assigned to study groups using sequentially numbered, sealed, opaque envelopes. Participants were masked to the sequence.^24^

### Study Outcomes

The primary outcome was the participants’ preference for optimistic vs pessimistic framing. This was assessed once, after the second video, in response to the binary question of whether a participant preferred the first or the second video. Complementary to the primary outcome, participants indicated a general framing preference, ie, their preferred level of optimism in the framing of prognostic information (7-point scale: 1 [not at all optimistic] to 7 [very optimistic]).

The following secondary outcomes were assessed. At baseline and after each video, participants’ state anxiety (STAI-SKD^29^), ie, anxiety as a transient response to a stimulus, was assessed as framing effect on an emotional level. A higher sum score (range, 5 to 20) indicated a higher level of state anxiety. Other secondary outcomes were only assessed after the respective first video. Participants rated the physician’s overall impression (from 1 [poor] to 5 [very good]), physician professionalism (sum score range, 7-35), and physician compassion (sum score range, 5-50). Physician professionalism was assessed using a 7-item questionnaire adapted from the General Medical Council (GMC) patient questionnaire.^30^ The selection of items was adapted from Tanco et al.^22,31^ Physician compassion was measured with the Physician Compassion Questionnaire^32^ (original scale inverted) also adapted from Tanco et al.^22,31^ Higher scores indicated higher levels of professionalism and compassion. Participants’ perceptions of prognostic communication (satisfaction with framing, level of information about the prognosis, preparedness for decision making) and prognostic expectations (favorability of the prognosis, optimism, and hope for the infant’s future) were assessed using individually tailored questions. For each response, fully verbalized 7-point rating scales (from 1 [not at all] to 7 [very much or completely]) with a verbal equivalent for each scale point were used. Recall accuracy of the numerical estimates for survival and impairment was assessed by percentages selected by the participants. A choice of percentages between 0 and 100% in increments of 10 (for survival) or 25 (for impairment) was requested.

### Statistical Analysis

Sample size calculation aimed to detect a preference odds (ratio of preference for optimistic vs pessimistic framing) of 3:2 with 80% power by a period-adjusted analysis that accounted for 1 or 2 responding parents, respectively. This resulted in 215 single parents or 153 parent couples.^24^ After a planned masked sample size reassessment based on responses from 144 parents in 90 families, we calculated a required sample size of 265 parents. End of individual recruitment was further defined as the time at which each eligible family, which had not been reached at the time of reassessment, had been contacted 5 times at 5 different times of day on 5 different days over a 5-week period. Data collection therefore ended 4 and a half months into the study when no more parents could be recruited from the eligible population.

Statistical analysis was performed using IBM SPSS Statistics 27 for Windows (IBM Corp). Analyses followed a modified intention-to treat approach. Participants who were randomized but did not start the study (ie, did not watch a single video) were excluded from the analysis. Standard descriptive statistics including means and medians, and proportions were calculated for all baseline and outcome variables. For outcome variables, appropriate effect estimates are reported along with the corresponding 95% CIs. For inferential statistics, all tests were 2-sided, and a *P* value < .05 was considered statistically significant. For all variables, the statistics have been adjusted for intrafamilial correlation (IFC), ie, the tendency of parents of the same infant to respond similarly. The IFC was quantified by the intraclass correlation coefficient (ICC) in percentage. The primary outcome was analyzed by fitting a marginal logistic regression model for correlated binary data to account for a period effect and the IFC.^24^

## Results

**Figure 1.**
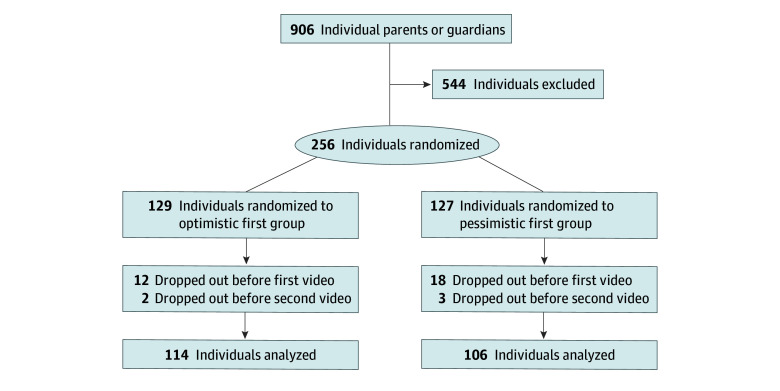
Participant Flow for the COPE-Trial

Of 906 individuals screened, 256 were randomized and 220 were included in the final analysis (Figure 1). Our sample included 142 female participants (64.5%), and 203 participants (92.3%) lived in a 2-parent household (Table 1). Most participants (44.1%) had 2 children, and their preterm infant had been in the NICU a mean (SD) 5.9 (2.8) years ago (range, 2.0-11.0 years). No participant discontinued study participation for elevated participation-related psychological distress or requested support by a research team member or a mental health professional.

**Table 1.** Baseline Characteristics of Individual Participants by Intervention Groups and in Total

Characteristics	Participants, No. (%)^a^		
	Optimistic first (n = 114)	Pessimistic first (n = 106)	Total (n = 220)
**General characteristics**			
Constellation of participating caregivers			
Both partners	60 (52.6)	54 (50.9)	114 (51.8)
Mothers only	44 (38.6)	41 (38.7)	85 (38.6)
Fathers only	10 (8.8)	11 (10.4)	21 (9.5)
Gender^b^			
Female	74 (64.9)	68 (64.2)	142 (64.5)
Male	40 (35.1)	38 (35.8)	78 (35.5)
Age at participation, mean (SD) [range], y			
Mothers	39.4 (5.2) [28.0-52.0]	38.7 (6.0) [25.0-56.0]	39.1 (5.6) [25.0-56.0]
Fathers	43.0 (7.0) [32.0-60.0]	42.4 (7.0) [30.0-58.0]	42.7 (6.9) [30.0-60.0]
**Sociocultural background**			
Migration experience			
Living in Germany since birth^c^	92 (80.7)	94 (88.7)	186 (84.5)
Born elsewhere, immigrated to Germany	22 (19.3)	12 (11.3)	34 (15.5)
Germany as country of identification^d^	98 (86.0)	91 (86.7)	189 (86.3)
Multilingual	24 (21.1)	15 (14.2)	39 (17.7)
German language acquisition			
First language	91 (79.8)	92 (86.8)	183 (83.2)
Second language	5 (4.4)	5 (4.7)	10 (4.5)
Foreign language	18 (15.8)	9 (8.5)	27 (12.3)
Religiosity, mean (SD) score [range]^e^	2.2 (1.1) [1.0-5.0]	2.7 (1.0) [1.0-5.0]	2.5 (1.1) [1.0-5.0]
**Education, occupation, and medical expertise**			
Basic education			
Basic general education	1 (0.9)	2 (1.9)	3 (1.4)
Medium general or vocational education	29 (25.4)	28 (26.4)	57 (25.9)
General (technical) university entrance qualification	83 (72.8)	75 (70.7)	158 (71.8)
Other	1 (0.9)	1 (0.9)	2 (0.9)
Professional education			
No or noncompleted vocational training or studies	4 (3.5)	3 (2.8)	7 (3.2)
Vocational training (in-company or school-based)	47 (41.2)	47 (44.3)	96 (42.7)
University (of applied sciences) degree^f^	62 (54.4)	54 (50.9)	126 (52.7)
Other	1 (0.9)	2 (1.9)	3 (1.4)
Occupation			
Student	0	1 (0.9)	1 (0.5)
Employee	76 (66.7)	72 (67.9)	148 (67.3)
Civil servant	12 (10.5)	14 (13.2)	26 (11.8)
Self-employed	16 (14.0)	8 (7.5)	24 (11.8)
Full-time at home for children, househusband or housewife	5 (4.4)	10 (9.4)	15 (6.8)
Unemployed or job-seeking	2 (1.8)	0	2 (0.9)
Other	3 (2.6)	1 (0.9)	4 (1.8)
Medical expertise (by education or profession)	26 (22.8)	22 (20.8)	48 (21.8)
NICU experience (professional)	3 (2.6)	1 (0.9)	4 (1.8)
Family and premature infant			
Household			
Single-parent	4 (3.5)	5 (4.7)	9 (4.1)
2-parent	106 (93.0)	97 (91.5)	203 (92.3)
>2 parents, patchwork	4 (3.5)	4 (3.8)	8 (3.6)
No. of children			
1	36 (31.6)	33 (31.1)	69 (31.4)
2	49 (43.0)	48 (45.3)	97 (44.1)
3	21 (18.4)	15 (14.2)	36 (16.4)
>3	8 (7.0)	10 (9.4)	18 (8.2)
Time since own NICU experience, mean (SD) [range], y	5.9 (2.8) [2.0-11.0]	5.8 (2.8) [2.0-11.0]	5.9 (2.8) [2.0-11.0]

Abbreviation: NICU, neonatal intensive care unit.

^a^ Due to the rounding of the relative numbers of each expression of a characteristic to one decimal place, their sum may not always add up to exactly 100%. Characteristics of participating partners were considered separately.

^b^ In the self-reported data, no one selected the third category, “diverse.”

^c^ Includes individuals with migration background where previous generations may have had a first-person migration experience.

^d^ Data on the country of identification were missing for 1 participant in the pessimistic first group (219 total participants; 105 participants in pessimistic first group).

^e^ Participants could rate themselves as religious or devout on a 5-point scale from not at all (1), a little, moderately, strongly, or very strongly (5).

^f^ Summarizes participants with a bachelor’s, master’s, and a doctoral degree.

### Primary Outcome

Participants preferred optimistic over pessimistic framing (196 of 220 [89.1%] vs 24 of 220 [10.9%]). The preference probability for optimistic framing was estimated to be 92% (95% CI, 86%-95%) after model-based adjustment for presentation order and IFC. The respective preference odds was 11.0 (95% CI, 6.28-19.10; *P* < .001).

The preference for optimistic framing was more pronounced when presented second than when presented first (adjusted preference probability: optimistic framing second, 96% [95% CI, 90%-99%] vs first, 82% [95% CI, 74%-89%]; preference odds, 5.41 [95% CI, 1.77-16.48]; *P* = .003).

### Secondary Outcomes

Participants who preferred the optimistic framing video were more likely to have a general preference for optimism (adjusted mean: preference for optimistic framing, 4.72 [95% CI, 4.62-4.83] vs pessimistic framing, 3.79 [95% CI, 3.49-4.10]; adjusted mean difference, 0.93 [95% CI, 0.61-1.25]; *P* < .001).

**Figure 2.**
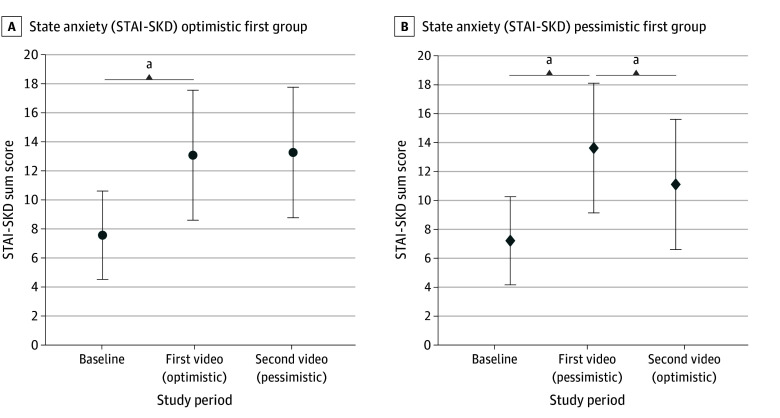
State Anxiety (STAI-SKD) Scores for Before and After Video Viewings ^a^Significant results (*P* < .05).

Baseline state anxiety scores were similar in both groups (adjusted mean [SD]: optimistic, 7.29 [3.04] vs pessimistic, 7.63 [3.04]; adjusted mean difference, −0.34 [−1.18 to 0.49]; *P* = .42). In response to the first video, with both optimistic and pessimistic framing, participants’ state anxiety scores increased equally from baseline (adjusted mean [SD]: optimistic first, 13.13 [4.47] vs pessimistic first, 13.68 [4.47]; *P* < .001 for each). When pessimistic framing followed optimistic framing, state anxiety scores remained unchanged (adjusted mean [SD]: optimistic first, 13.13 [4.47] vs pessimistic second, 13.32 [4.49]; *P* = .54) (Figure 2A). In contrast, when optimistic framing followed pessimistic framing, state anxiety scores decreased (adjusted mean [SD]: pessimistic first, 13.68 [4.47] vs optimistic second, 11.17 [4.49]; *P* < .001) (Figure 2B).

When comparing optimistic vs pessimistic framing, participants rated their overall impression of the physician as more positive (adjusted mean [SD], optimistic 3.79 [0.97] vs pessimistic 2.55 [0.97]; adjusted mean difference, 1.24 [95% CI, 0.98 to 1.50]; *P* < .001) (eFigure 1 in Supplement 1). They also rated the physician as more professional (adjusted mean [SD]: optimistic, 26.57 [5.07] vs pessimistic, 19.93 [5.07]; adjusted mean difference, 6.64 [95% CI, 5.29 to 8.00]; *P* < .001) and more compassionate (adjusted mean [SD]: optimistic, 34.48 [9.36] vs pessimistic, 14.87 [9.36]; adjusted mean difference, 19.61 [95% CI, 17.06 to 22.17]; *P* < .001) (eFigure 1 in Supplement 1). The ICC was 7.3% for overall impression, 1.7% for professionalism, and 10.3% for compassion.

With optimistic framing, participants were more satisfied with the prognostic communication style (4.83 [1.48] vs 2.81 [1.48]) (Table 2). They felt better informed about the prognosis (4.99 [1.64] vs 3.86 [1.64]) and better prepared for SDM (3.79 [1.53] vs 2.60 [1.53]) as surrogates for their child. Participants also perceived the conveyed prognosis as more favorable (3.23 [1.15] vs 2.48 [1.15]). They were more optimistic about the infant’s survival (4.42 [1.30] vs 3.64 [1.30]) and nonimpairment (3.41 [1.25] vs 2.46 [1.25]), and more hopeful for the infants’ future (4.28 [1.48] vs 3.28 [1.48]).

**Table 2.** Effects of Optimistic vs Pessimistic Framing on Parental Perceptions Assessed After Presentation of the First Video

Outcomes	Mean (SD) scores^a^		Comparison of framing effects		ICC,%
	Optimistic framing (n = 114)	Pessimistic framing (n = 106)	Mean difference (95% CI)	*P* value	
Perception of prognostic communication					
Satisfaction with prognostic framing	4.83 (1.48)	2.81 (1.48)	2.02 (1.63-2.42)	<.001	0
Level of information about prognosis	4.99 (1.64)	3.86 (1.64)	1.13 (0.70-1.57)	<.001	0
Preparedness for decision-making	3.79 (1.53)	2.60 (1.53)	1.19 (0.76-1.62)	<.001	23.9
Prognostic expectations					
Favorability of prognosis	3.23 (1.15)	2.48 (1.15)	0.75 (0.43-1.08)	<.001	22.6
Optimism					
Concerning survival	4.42 (1.30)	3.64 (1.30)	0.78 (0.41-1.15)	<.001	33.9
Concerning nonimpairment	3.41 (1.25)	2.46 (1.25)	0.94 (0.60-1.29)	<.001	19.1
Hope	4.28 (1.48)	3.28 (1.48)	1.01 (0.61-1.40)	<.001	2.2

Abbreviation: ICC, intraclass correlation coefficient.

^a^ Higher scores indicate a more pronounced expression of the respective effect (range, 1-7). In the model, optimistic framing was used as reference category.

**Figure 3.**
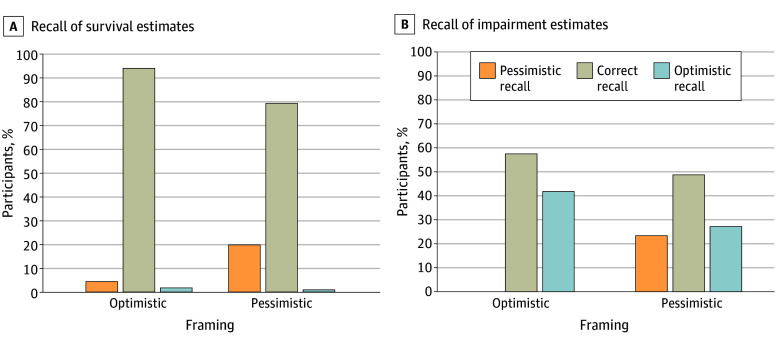
Recall of Numerical Outcome Estimates Optimistic framing included 114 parents after presentation of the first video; pessimistic framing, 106 parents.

Figure 3A and Figure 3B visualize the proportion of participants whose recall of conveyed outcome estimates was correct, optimistic (overestimation of survival, underestimation of impaired survival), or pessimistic (underestimation of survival, overestimation of impaired survival). With optimistic framing, the odds of correct recall of conveyed estimates were higher for survival (odds ratio, 4.00; 95% CI, 1.64-9.79; *P* = .002). A similar but nonsignificant trend was observed for impairment (odds ratio, 1.50; 95% CI, 0.85-2.63; *P* = .16). With both framing variants, when deviant, recall of survival estimates was more likely to be pessimistic than optimistic (Figure 3A). With pessimistic framing, however, this trend was more pronounced (odds ratio, 8.40; 95% CI, 0.63-112.42; *P* = .11), although the result was not statistically significant. In contrast, when deviant, recall of impairment estimates was rather optimistic than pessimistic with both framing variants (Figure 3B). However, solely with pessimistic framing it was in part pessimistic. The trend for pessimistic recall of impairment estimates was more pronounced with pessimistic framing (*P* for trend < .001).

## Discussion

The COPE-Trial provides evidence that parents of very preterm infants may prefer a more optimistic view of the outcome of a serious complication. This is consistent with previous findings that parents prefer an overall optimistic view of their child’s prognosis and appreciate physicians who communicate the risk of a poor outcome while acknowledging the chances of a good outcome.^33^ Previous studies have shown that neonatologists often have a more pessimistic view of an infant’s prognosis than parents^4,7,11,34,35^ and are perceived by parents to be more pessimistic in their prognostic communication.^11,33,34,36^ Parents value honest and realistic communication about their child’s prognosis, but appreciate that positive aspects are also emphasized.^12,14,33,37^ The level of optimism that parents consider optimal seems to be the key. Parents seem to prefer positive language, whereas what may be taken as excessive optimism or the sugarcoating of information is likely to be perceived as threatening to the parent-doctor relationship.^12,38,39,40,41^ Our study results are consistent with these previous findings in that parents prefer an optimistic framing when communicating prognostic predictions.

In terms of framing preference, we found a sequence effect in favor of the respective second framing variant in both groups. This finding may be interpreted as a recency effect.^42^ A similar sequence effect for preference has been observed in previous video-based communication studies in adult oncology, including one by Tanco and colleagues.^22^ This effect may be due to an increased receptivity to multiple layers of communication and the critical information itself when a serious message is repeated. However, given the complexity and multidimensionality of communication, it is conceivable that this effect may also be attributable to characteristics of the parent-physician interaction, including the emotional response to the delivery of a serious message.

The latter assumption is supported by the observation of a sequence dependence not only for the framing preference but also for the emotional response to optimistic and pessimistic framing. In our study, the first communication of a serious message elicited a pronounced increase in the participants’ state anxiety. This is consistent with the findings of previous video-based communication studies in adult oncology^23,43^ and confirms an authentic emotional response to communication under experimental conditions for the NICU setting.^44^ Consistent with Zwingmann and colleagues^43^ and Porensky and Carpenter,^23^ we found an effect of the physician communication style on the recipient’s emotional response. But in our study, the framing-dependent difference in response was only substantial when the message was delivered a second time and with the respective opposite framing. We suspect that this may be the result of an emotional reaction. When the message was repeated with optimistic framing, state anxiety decreased substantially. However, when it was repeated with pessimistic framing, it remained almost unchanged.

Message framing has been shown to influence the perception of information and SDM in the NICU setting.^17,19,20^ There is a growing body of evidence supporting the view that the process of communication, rather than the information itself, deserves most attention when counseling parents.^18^ This study supports this notion, suggesting that framing influences perceptions of the attending physician and of essential components of the SDM process, including satisfaction with communication.^22,23^ Our study also confirms for the NICU setting, that optimistic framing affects the parents’ prognostic expectations and the physicians’ and parents’ shared understanding of a prognosis. Framing causes medical facts to be perceived differently. This seems to be particularly true for the parents’ assessment of the risk of the very preterm infant to retain impairment. This observation can be well explained by the optimism bias. Very serious prognoses are perceived as less serious than they really are.^45,46^ A potentially overly optimistic view of the infant’s neurodevelopment with the preferred optimistic framing may be addressed by specific strategies. These might include the repetition of prognostic information in the course^7,12,47,48^ or explicitly supplementing the potential positive outcomes conveyed with risks and potential negative outcomes in the sense of a mixed framing.^23^ Additional written, visual, or audiovisual materials could be an appropriate measure to reinforce verbal information and to enhance parental understanding.^16,41,49,50,51,52^ However, it should also be recognized that optimizing prognostic recall, especially of impairment estimates, may not be necessary. Impairment estimates appear to be less meaningful outcomes to NICU parents than survival estimates.^7,9,53,54^ Moreover, parents generally tend to be more positive about their child’s prognosis than physicians. A hopeful and optimistic view of the child’s future by parents can be realistic even when the prognosis is poor. Recent studies demonstrate that hope and realism are not mutually exclusive in the context of understanding essential information in the NICU.^7,47,55^ A rather positive view on the future may not be harmful in the first instance, as hopes are broad and can change in the course.^56,57,58^

Conclusions for practice should be drawn with caution, mainly because these results are drawn from simulated conversations outside of everyday clinical practice. In addition, it is difficult to draw conclusions from this general approach to individual communication. However, we believe that clinicians may find a more optimistic framing reassuring because it is likely to be in line with parents’ preferences and may lead to more realistic expectations about prognosis while maintaining parents’ hopes.

### Limitations

This study had several limitations. It is likely that the course and outcome of their own child, as well as parents’ personal characteristics or emotions, may have influenced participants’ responses.^4,12,19,31,59^ Enrollment was lower than expected, and generalizability is limited by the single-center design and underrepresentation of parents groups whose preference may differ (parents with mental health concerns, bereaved parents, parents from racial and ethnic minority groups).^60,61,62^ In retrospect, parents of deceased infants may have preferred pessimistic framing. Video vignettes proved to be a challenging intervention as framing a message as optimistic or pessimistic is complex and multidimensional. The intention was to keep as many aspects of the videos standardized and to vary framing as a selected aspect of prognostic communication. Congruent with the framing as a variation on the verbal level of communication, a difference occurred on the nonverbal level, such as the neonatologist’s voice color, which includes vocal tone, pronunciation, resonance, and voice strength. We further recognize that the selected outcomes represent a simplification of a spectrum of possible outcomes, which may limit their meaningfulness to parents.^11^

## Conclusions

The COPE-Trial provides evidence that a large proportion of parents of very preterm infants may prefer optimistic prognostic communication. These results warrant further investigation in the clinical setting.

## Notes

Supplement 1. **eAppendix 1.** Scripts for Video Vignettes (English Translation) **eAppendix 2.** Scripts for Video Vignettes in German **eFigure 1.** Perception of Physician

Supplement 2.
Trial Protocol

Supplement 3.
Data Sharing Statement
