# Supplementary material for: Optimistic vs Pessimistic Message Framing in Communicating Prognosis to Parents of Very Preterm Infants: The COPE Randomized Clinical Trial
Source: JAMA Netw Open. 2024 Feb 23;7(2):e240105. doi: 10.1001/jamanetworkopen.2024.0105 (PMC10891472; doi:10.1001/jamanetworkopen.2024.0105)
Supplement: Supplement 1. — eAppendix 1. Scripts for Video Vignettes (English Translation) eAppendix 2. Scripts for Video Vignettes in German eFigure 1. Perception of Physician [file jamanetwopen-e240105-s001.pdf]

Supplemental Online Content

Forth FA, Hammerle F, König J, et al. Effects of optimistic vs pessimistic message framing in communicating prognosis to parents of very preterm infants. *JAMA Netw Open.* 2024;7(2):e240105. doi:10.1001/jamanetworkopen.2024.0105

eAppendix 1. Scripts for Video Vignettes (English Translation)

eAppendix 2. Scripts for Video Vignettes in German

eFigure 1. Perception of Physician

This supplemental material has been provided by the authors to give readers additional information about their work.

© 2024 Forth FA et al. *JAMA Network Open.*

eAppendix 1. Scripts for Video Vignettes (English Translation)

a | INTRODUCTION: EXPLANATORY VIDEO

VOICE OVER The following scene is set at the University Medical Center Mainz. In the Neonatal Intensive Care Unit. The setting is a counselling situation: The parents of a premature infant are receiving a severe diagnosis.

After this video, we will kindly ask you to answer a few questions.

It is the third day of life of a very immature premature baby. Luisa. A girl. She was born after 23 weeks and 5 days of pregnancy. Weighing as little as 580 grams. Such an early birth carries a high risk of complications. One of those typical for premature infants is for example: a cerebral hemorrhage. Luisa's parents have already talked to the attending physicians: About premature birth and possibly arising complications thereof.

As one of these sets in, they are contacted immediately.

Link to the video on YouTube: https://youtu.be/ha4GuVzwAN4

b | OPTIMISTIC VIDEO

PHYSICIAN: (*very calmly, without rush*) Thank you for coming on such short notice.

MOTHER: *(worried/anxiously)* What happened? How is Luisa?

PHYSICIAN: (*slowly and calmly*) She is stable at the moment. It is not life-threatening. However, ultra-sound showed a (cerebral hemorrhage) bleeding in her brain. Hence the call. As you may remember: We have talked about possible complications stemming from premature birth. Unfortunately, we've now diagnosed one with your daughter.

FATHER: (*pulling himself together*) A bleeding in her brain. But how?

PHYSICIAN: Within the brain of a premature infant there are areas especially prone to bleeding. The exact mechanisms – of how those bleedings arise – are unfortunately still widely unknown.

Here you can see the bleeding affecting much of the right cerebral hemisphere. The white area here – that's the extension of the bleeding.

MOTHER: *(after a while)* And what … what does that mean now for Luisa?

PHYSICIAN: A bleeding (cerebral hemorrhage) of this extension. That is a severe complication. We have to assume (It must be assumed): It won't be without consequences for Luisa's further treatment. And for her development (in general). There is comparable cases: Around half of the children with a bleeding of this kind survives. And of the surviving children, half again do not retain a noticeable impairment. The prognosis for affected children is therefore (very) serious. In similar cases/in cases as such, parents and physicians often decide to no longer uphold intensive medical treatment. The exact consequences for Luisa – as of now, are not easy to predict. It is however possible that the affects will be minor/she will only be minorly/mildly affected.

FATHER: *(grieving, sad)* But … is there no treatment? (Can it/this not be treated somehow?)

PHYSICIAN: I wish there were one, but no. A bleeding (cerebral hemorrhage) as such can unfortunately not be treated to date. I can only speculate at this point. There might be new possibilities for therapy in the future. There has been, for example, preliminary testing with stem cells, in order to support (enhance) recreation/regeneration. This treatment though is not yet available in the here and now.

MOTHER*: (shivering voice)* What will happen now to Luisa? What should/shall/can we do?

PHYSICIAN: Nobody know what the future might hold. Lots of children do not survive situations like this. Most of them/those who do only with severe impairments/retain severe impairments.

© 2024 Forth FA et al. *JAMA Network Open.*

I do, however, know children who after a severe bleeding of this kind, have developed normally.

MOTHER / FATHER: (*crying*)

PHYSICIAN: (*equally moved/saddened/empathetic*) I will give you (two) a moment, okay? I suggest we will sit down together again later. Then we can talk about our next steps.

Link to the video on Youtube: https://youtu.be/MpSc5T31z68

c | PESSIMISTIC VIDEO

PHYSICIAN: (*very calmly, without rush*) Thank you for coming on such short notice.

MOTHER: *(worried/anxiously)* What happened? How is Luisa?

PHYSICIAN: (*slowly and calmly*) She is stable at the moment. It is not life-threatening. However, ultra-sound showed a (cerebral hemorrhage) bleeding in her brain. Hence the call. As you may remember: We have talked about possible complications stemming from premature birth. Unfortunately, we've now diagnosed one with your daughter.

FATHER: (*pulling himself together*) A bleeding in her brain. But how?

PHYSICIAN: Within the brain of a premature infant there are areas especially prone to bleeding. The exact mechanisms – of how those bleedings arise – are unfortunately still widely unknown.

What you see here is a severe bleeding affecting much of the right cerebral hemisphere. The white area here – that's the extension of the bleeding.

MOTHER: *(after a while)* And what … what does that mean now for Luisa?

PHYSICIAN: A bleeding (cerebral hemorrhage) of this extension. That is a grievous complication. We have to assume (It must be assumed): It won't be without consequences for Luisa's further treatment. And for her development (in general). There is comparable cases: Around half of the children with a bleeding of this kind do not survive. And of the surviving children, half again retain a noticeable impairment. The prognosis for affected children is therefore (very) serious. In similar cases/in cases as such, parents and physicians often decide to stop (end) the intensive medical treatment. The exact consequences for Luisa – as of now, are hard to predict. It's probable that she will not be completely healthy or that she will not survive.

FATHER: *(grieving, sad)* But … is there no treatment? (Can it/this not be treated somehow?)

PHYSICIAN: I wish there were one, but no. The bleeding (cerebral hemorrhage) as such can unfortunately not be treated. Only the acute affects, i.e. on her (bodily) circulation. Or we can alleviate her symptoms. But we cannot influence the cause of the diagnosis itself. The process cannot be reversed. (It is impossible to reverse the process.)

MOTHER*: (shivering voice)* What will happen now to Luisa? What should/shall/can we do?

PHYSICIAN: Nobody know what the future might hold. Lots of children do not survive situations like this. Most of them/those who do only with severe impairments/retain severe impairments. I wish, the prognosis were different.

MOTHER / FATHER: (*crying*)

PHYSICIAN: (*equally moved/saddened/empathetic*) I will give you (two) a moment, okay? I suggest we will sit down together again later. Then we can talk about our next steps.

Link to the video on Youtube: https://youtu.be/_ZjiCCoLP9c

© 2024 Forth FA et al. *JAMA Network Open.*

eAppendix 2. Scripts for Video Vignettes in German

a | EINLEITUNG: ERKLÄRFILM

VOICE OVER Die nachfolgende Szene spielt in der Universitätsmedizin Mainz. Auf der Intensivstation der Neonatologie. Rahmen bildet ein Beratungsgespräch: Die Eltern eines Frühgeborenen bekommen darin eine schwerwiegende Diagnose und übermittelt.

Nach dem Video möchten wir Sie bitten, uns ein paar Fragen zu beantworten.

Es ist der 3. Lebenstag eines sehr unreifen Frühgeborenen. Luisa. Ein Mädchen. Sie wurde nach 23 Schwangerschaftswochen und 5 Tagen geboren. Mit nur 580 Gramm. Eine so frühe Geburt birgt große Risiken für Komplikationen. Für Frühgeborene sehr typisch ist zum Beispiel: Eine Hirnblutung. Luisas Eltern haben mit den behandelnden Ärztinnen bereits gesprochen: Über die Frühgeburtlichkeit und mögliche, damit einhergehende Komplikationen. Als es dazu kommt, werden sie unmittelbar kontaktiert.

Link to the video on Youtube: https://youtu.be/haPSDlELpew

b | OPTIMISTISCHES VIDEO

ÄRZTIN: (*sehr ruhig, ohne Hektik*) Danke, dass Sie so schnell gekommen sind.

MUTTER: *(sorgenvoll)* Was ist denn passiert? Wie geht es Luisa?

ÄRZTIN: (*langsam und ruhig*) Im Moment ist sie stabil. Keine Lebensgefahr. Allerdings haben wir bei einer Ultraschall-Untersuchung eine Hirnblutung diagnostiziert. Deshalb der Anruf. Sie erinnern sich: Vorgestern haben wir ja bereits über mögliche Komplikationen bei einer Frühgeburt gesprochen. Jetzt haben wir eine solche leider bei Ihrer Tochter festgestellt.

VATER: (*fasst sich*) Hirnblutung? Wie ist das passiert?

ÄRZTIN: Im Gehirn von sehr unreifen Frühgeborenen gibt es Bereiche, die besonders anfällig für Blutungen sind. Der genaue Mechanismus – wie solche Blutungen im Gehirn entstehen – das alles ist uns leider noch nicht bekannt.

Sie sehen hier die Blutung. Sie betrifft den Großteil der rechten Hirnhälfte. Der weiße Bereich hier – das ist die ausgedehnte Blutung.

MUTTER: *(nach einer Weile)* „Und was … Was heißt das jetzt für Luisa?

ÄRZTIN: Eine Hirnblutung von diesem Ausmaß. Das ist eine ernstzunehmende Komplikation. Man muss davon ausgehen: Das ist nicht folgenlos für Luisas weitere Behandlung. Und für ihre Entwicklung. Es gibt vergleichbare Fälle: Etwa die Hälfte der Kinder mit einer solchen Blutung überlebt. Und von den Überlebenden behält wiederum die Hälfte keine deutliche Behinderung. Die Prognose ist also ernst bei derart betroffenen Kindern. In vergleichbaren Fällen entscheiden Eltern und Ärztinnen häufig nicht länger intensivmedizinisch zu behandeln.

Die genauen Folgen für Luisa – die sind zum jetzigen Zeitpunkt zwar nur schwer vorhersehbar. Es ist jedoch auch möglich, dass sie nur wenig betroffen ist.

VATER: *(betroffen)* Aber … Kann man das nicht irgendwie behandeln?

ÄRZTIN: Ich wünschte es wäre so. Nein. Eine solche Hirnblutung kann heute leider noch nicht behandelt werden. Ich kann im Moment nur spekulieren. Vielleicht gibt es zukünftig neue Therapie-Möglichkeiten. Zum Beispiel wurden schon erste Versuche mit Stammzellen gemacht. Um das Gehirn zu unterstützen bei der Regeneration. Diese Behandlung aber – die ist im Hier und Jetzt noch nicht verfügbar.

MUTTER*: (zittrige Stimme)* Was wird denn jetzt aus Luisa? Was machen wir bloß?

© 2024 Forth FA et al. *JAMA Network Open.*

ÄRZTIN: Niemand weiß, was die Zukunft bringt. Einige Kinder überleben solche Situationen nicht. Andere nur mit deutlichen Einschränkungen. Ich kenne aber auch Kinder, die sich nach so einer schweren Hirnblutung normal entwickelt haben.

MUTTER / VATER: (*weinen)*

ÄRZTIN: (*ebenfalls ergriffen*) Ich gebe ich Ihnen einen Moment Zeit. Ja? Mein Vorschlag: Wir setzen uns nachher nochmal zusammen. Dann besprechen wir unser weiteres Vorgehen.

Link to the video on Youtube: https://youtu.be/xX8DkaOCULA

c | PESSIMISTISCHES VIDEO

ÄRZTIN: (*sehr ruhig, ohne Hektik*) Danke, dass Sie so schnell gekommen sind.

MUTTER: *(sorgenvoll)* Was ist denn passiert? Wie geht es Luisa?

ÄRZTIN: (*langsam und ruhig*) Im Moment ist sie stabil. Keine Lebensgefahr. Allerdings haben wir bei einer Ultraschall-Untersuchung eine Hirnblutung diagnostiziert. Deshalb der Anruf. Sie erinnern sich: Vorgestern haben wir ja bereits über mögliche Komplikationen bei einer Frühgeburt gesprochen. Jetzt haben wir eine solche leider bei Ihrer Tochter festgestellt.

VATER: (*fasst sich*) Hirnblutung? Wie ist das passiert?

ÄRZTIN: Im Gehirn von sehr unreifen Frühgeborenen gibt es Bereiche, die besonders anfällig für Blutungen sind. Der genaue Mechanismus – wie solche Blutungen im Gehirn entstehen – das alles ist uns leider noch nicht bekannt.

Sie sehen hier die schwere Blutung. Sie betrifft den Großteil der rechten Hirnhälfte. Der weiße Bereich hier – das ist die ausgedehnte Blutung.

MUTTER: *(nach einer Weile)* „Und was … Was heißt das jetzt für Luisa?

ÄRZTIN: Eine Hirnblutung von diesem Ausmaß. Das ist eine schwerwiegende Komplikation. Man muss davon ausgehen: Das ist nicht folgenlos für Luisas weitere Behandlung. Und für ihre Entwicklung. Es gibt vergleichbare Fälle: Etwa die Hälfte der Kinder mit einer solchen Blutung überlebt nicht. Und von den Überlebenden behält wiederum die Hälfte eine deutliche Behinderung. Die Prognose ist also ernst bei derart betroffenen Kindern. In vergleichbaren Fällen entscheiden Eltern und Ärztinnen häufig, die intensiv-medizinische Behandlung zu beenden. Die genauen Folgen für Luisa – die sind zum jetzigen Zeitpunkt nur schwer vorhersehbar. Wahrscheinlich ist: Sie ist später nicht ganz gesund oder überlebt nicht.

VATER: *(betroffen)* Aber … Kann man das nicht irgendwie behandeln?

ÄRZTIN: Ich wünschte es wäre so. Nein. Die Hirnblutung an sich kann leider nicht behandelt werden. Nur die akuten Auswirkungen. Zum Beispiel auf den Kreislauf. Oder die Symptome, die können wir lindern. Den Befund an sich aber, den können wir nicht ursächlich beeinflussen. Das Geschehen lässt sich nicht rückgängig machen.

MUTTER*: (zittrige Stimme)* Was wird denn jetzt aus Luisa? Was machen wir bloß?

ÄRZTIN: Niemand weiß, was die Zukunft bringt. Viele Kinder überleben solche Situationen nicht. Die meisten nur mit deutlichen Einschränkungen. Ich wünschte, die Prognose wäre anders.

MUTTER / VATER: (*weinen)*

ÄRZTIN: (*ebenfalls ergriffen*) Ich gebe ich Ihnen einen Moment Zeit. Ja? Mein Vorschlag: Wir setzen uns nachher nochmal zusammen. Dann besprechen wir unser weiteres Vorgehen.

Link to the video on Youtube: https://youtu.be/K-SpGXlQawk

© 2024 Forth FA et al. *JAMA Network Open.*

eFigure 1. Perception of Physician

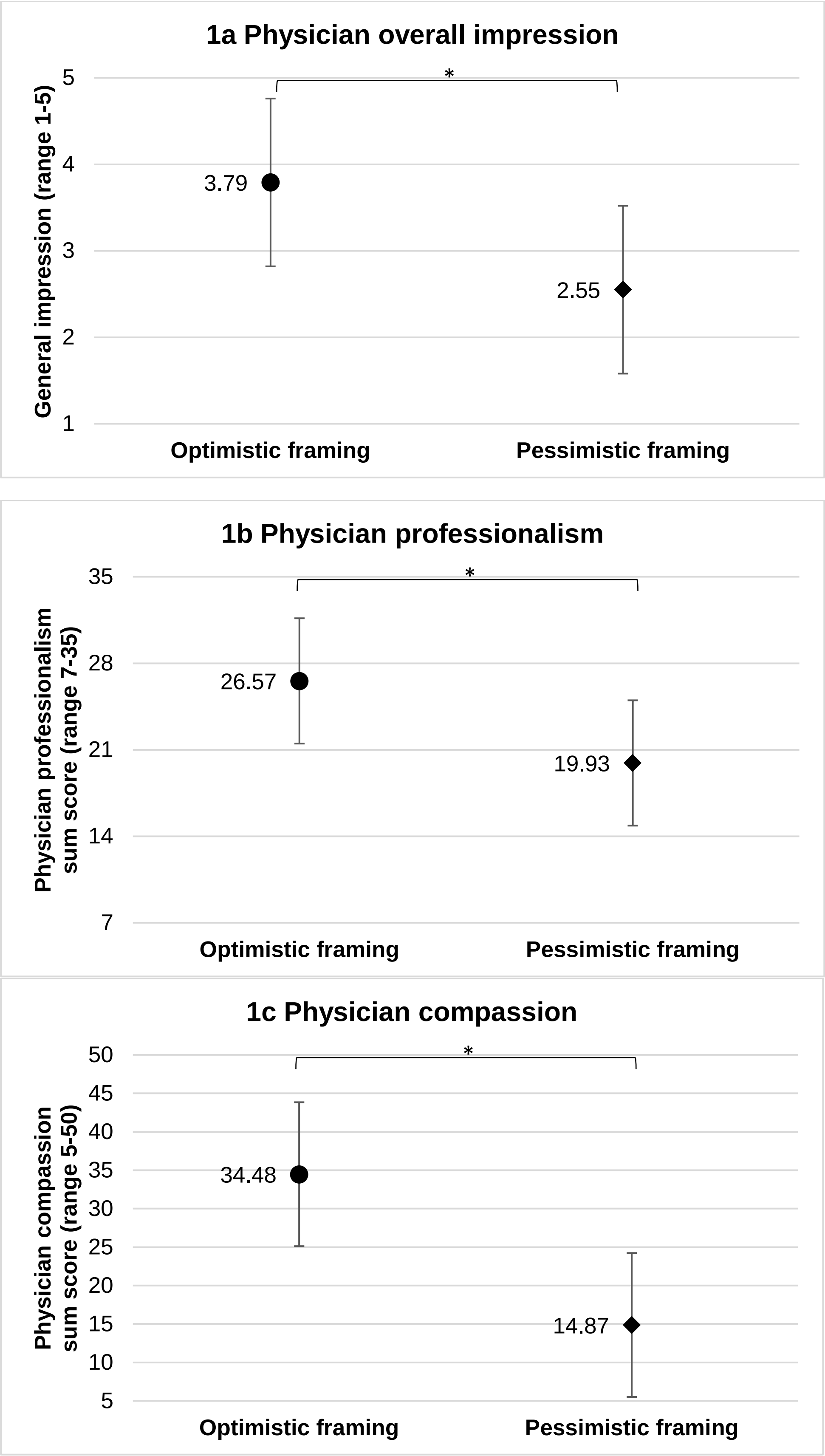

eFigure 1 Perception of physician

eFigures 1a,1b and 1c compare scores (mean±SD) for physician overall impression, professionalism and compassion with optimistic (n=114) versus pessimistic (n=106) framing. Significances are indicated with *.

© 2024 Forth FA et al. *JAMA Network Open.*
