# Supplementary material for: Optimistic vs Pessimistic Message Framing in Communicating Prognosis to Parents of Very Preterm Infants: The COPE Randomized Clinical Trial
Source: JAMA Netw Open. 2024 Feb 23;7(2):e240105. doi: 10.1001/jamanetworkopen.2024.0105 (PMC10891472; doi:10.1001/jamanetworkopen.2024.0105)
Supplement: Supplement 2. — Trial Protocol [file jamanetwopen-e240105-s002.pdf]

Scientific study protocol *(translated from German)*

I. General information

1. **List of abbreviations**

   GA - Gestational Age
   BW - Birth Weight
   NICU - Neonatal Intensive Care Unit

2. **Date and version**
   05.02.2021, final version 3.0 as approved by the local Ethics Committee of the Medical Association of Rhineland-Palatinate (Ethik-Kommission der Landesaerztekammer Rheinland-Pfalz), Mainz, Germany, on 01.04.2021.

3. **Title of the research project**
   COMMUNICATING PROGNOSIS TO PARENTS IN THE NEONATAL ICU: **O**PTIMISTIC VS. **PE**SSIMISTIC

   Acronym
   COPE

4. **Responsible study director / contact person**

Dr. med. André Kidszun, M.A.
Division of Neonatology
Center for Pediatric and Adolescent Medicine
University Medical Center of the Johannes Gutenberg-University Mainz
Langenbeckstrasse 1
55131 Mainz
Tel.: +49 (0) 6131 17-5892, Fax: +49 (0) 6131 17-3477
E-mail: andre.kidszun@unimedizin-mainz.de

Participating scientists

Cand. med. Fiona Antonia Forth, Univ.-Prof. Dr. med. Eva Mildenberger
Division of Neonatology, Center for Pediatric and Adolescent Medicine,
University Medical Center of the Johannes Gutenberg-University Mainz

Dr. Jochem König, Univ.-Prof. Dr. med. et med. univ. Michael S. Urschitz, EU-M.Sc.
Division of Pediatric Epidemiology, Institute of Medical Biostatistics, Epidemiology and Informatics (IMBEI), University Medical Center of the Johannes Gutenberg-University Mainz, Mainz, Germany

Philipp Neuweiler, M.A. in Media Dramaturgy
Research assistant at the Journalistisches Seminar, Johannes Gutenberg-University Mainz, Mainz, Germany

Dipl.-Psych. Florian Hammerle
Department of Pediatric and Adolescent Psychiatry and Psychotherapy, University Medical Center of the Johannes Gutenberg-University Mainz, Mainz, Germany

Responsible sponsor: University Medical Center Mainz

5. **Study type**
Single-center, double-blind, randomized-controlled study in parallel-group design

6. **Location of the research project**
Center for Pediatric and Adolescent Medicine
University Medical Center of the Johannes Gutenberg-University Mainz
Langenbeckstraße 1
55131 Mainz

7. **The research project is financially supported by the following third parties**

DFG Research Training Group "Life Sciences - Life Writing"
Funding code 2015/2

II. Justification of the research project

1. Background

When decisions need to be made in medicine, shared decision making is the gold standard of communication between physicians and patients. The declared aim is to enable patients or their representatives to make the best possible, jointly responsible (treatment) decision together with physicians through detailed, non-directive, non-prejudicial consultation. This type of decision-making is also sought by physicians in neonatology.[1] Beyond participatory decision-making, there is a recommendation in the context of neonatology, e.g. in antenatal consultations, to go one step further and strive for personalized decision-making in the case of impending premature birth.[2]

The birth of a very immature preterm infant is challenging even for experienced physicians due to the associated medical uncertainties, ethical controversy, and the need for timely interventions.[3] In addition, the setting of a neonatal intensive care unit (NICU) places high demands on parents.[4] Having just taken on their new role, they are expected to make vital decisions on behalf of their newborn (surrogate decision making), which have a significant impact on both their own and their child's future.
Ethically complex issues that are part of everyday life in the context of a NICU require a high level of communication. Only in this way the needs of all parties involved can be taken into account in consultation and decision-making processes. In the field of neonatal intensive care medicine, for this reason, more attention is nowadays paid to the process of decision-making than to achieving a very specific outcome.[5]

With the paradigm shift away from medical paternalism toward jointly responsible decisions in the sense of participatory decision-making, self-determination (autonomy) has gained importance as a core element of modern medicine. In this context, however, it is important to differentiate between two different forms of autonomy: absolute and relational autonomy.
Absolute autonomy allows patients (parents) to decide rationally, completely independent of the influence of their own emotions or those of others as an individual for themselves or in the best interests of the child. Physicians, as outsiders, are only allowed to (share) their factual knowledge and to inform. However, they must not take on an advisory role, so as not to color the individual's decision with their opinion.[6]

The philosophy of relational or contextual autonomy, on the other hand, postulates that every individual is in relationships with others and cannot make decisions independently of the influence of his or her environment. Rather, the individual achieves decision-making autonomy by linking his or her own rationality and emotionality with the needs of caregivers from the personal and medical, i.e., professional, environment, among others. Parents who make decisions on behalf of their children may, in the sense of relational autonomy, ask for or accept the advice of outsiders - for example, in the form of the personal interpretation of a situation or medical condition by the caring physician. Decisions are thus based on shared rational and emotional considerations.[6]

Absolute autonomy would consequently mean communicating facts in a completely neutral way. This is possible in principle, but absolute neutrality in the communication of important information is often difficult. Moreover, especially in the emotionally stressful context of the NICU, for parents, "neutral" facts require explanatory evaluation to optimize understanding of complex issues and to meet the needs of those affected. The way in which the facts are communicated inevitably influences the interpretation by those closest to them and thus their decision.[5] This makes the principle of relational autonomy a model that is feasible in practice and preferred by parents. It ultimately makes it possible to make authentic decisions with the support of professional staff.[4, 5]

In addition to preserving the autonomy of patients (parents), the application of the principles of care, beneficence, non-maleficence and justice is essential in medical consultation and decision-making processes.[7] In everyday clinical practice in general and in the context the NICU in particular, this means for medical staff to provide the best possible care for patients (here: premature infants), to be a support for their next of kin (parents) (care) and to make decisions with them in the best interests of the patients (beneficence). It is also important to provide open, honest and neutral information in order to give parents the information they need to make the most of their decision-making potential and to prevent harm (non-maleficence). In this context, harm can result, for example, from an overly vague prognosis that leads to a decision based on hoped-for outcomes rather than likely outcomes, resulting in patients receiving unnecessary therapies that are inappropriate to the real extent of the limitation of their prognosis.[8-10] Accordingly, with the patient's best interests in mind, vagueness of prognosis must be explicitly avoided. Since decisions made in the context of an inpatient stay of a very immature preterm infant are often vital ones, the communication of prognosis here particularly requires consideration of the principles just explained.

The communication of a prognosis should be an integral part of the communication between physicians and patients, as it enables them to make a realistic (treatment) decision appropriate to their situation.[11] Prognoses pose major challenges for parents and physicians alike. It is true that prognoses are based on medical experience and scientific knowledge. However, as a statement about the statistical or frequency probability of a (disease) course, they are always to a certain extent uncertain regarding the individual - including the possibility of the individual case deviating from the norm. For parents, the main challenge is to deal with the uncertainty that affects the future of their own premature baby and thus their entire environment, especially the family. For physicians, the uncertainty inherent in neonatology is a challenge for the following reasons: On the one hand, they are supposed to derive a personal prediction concerning the respective preterm infant from a frequent probability regarding the course of e.g., the diagnosis of a complication typical for preterm infants. On the other hand, it is the physician's task to communicate this outlook and the associated uncertainty to the parents. In doing so, physicians should draw a realistic picture, i.e., neither too optimistic nor too pessimistic. Prognostication therefore means making statements about a person's future that cannot be predicted with absolute certainty due to medical uncertainties and individual clinical courses.[8, 9] In this context, not only is the making of a prognosis a complex matter, but the communication of a prognosis becomes a complex undertaking due to the differing expectations of physicians and patients (and their parents). Successful doctor-parent discussions therefore require not only medical expertise on the part of the doctor, but also interpersonal competence to perceive the expectations of the parents and to behave accordingly.

Publications from specialties such as adult oncology, which are dedicated to questions in the context of communicating limiting prognoses, show that physicians initiate communication regarding prognosis and dominate it in terms of language in counseling and educational discussions.[12, 13] Prognoses are mostly formulated vaguely and supported by a focus on medical facts that are difficult for laypersons to understand.[9, 10, 12, 13] This obviously contradicts the desire of parents of critically ill children for clear communication of a concrete prognosis and explicit statements about the impact of the prognosis on the child's quality of life and the associated social environment. This type of parental desired communication succeeds mainly in delivering good news, whereas giving unfavorable prognoses is more challenging.[14] Previous studies also conclude that when communicating prognosis, care should be taken to balance concrete prognostication through honesty, as well as communicating hope in a realistic manner. Researchers postulate that this can be achieved by conveying medical empathy as well as the reassurance of non-abandonment and being available for queries.[15, 16]
The valuation of medical facts in the context of a consultation in clinical practice - whether intentional or not - is, as already mentioned, hardly avoidable. However, their influence on the consultation and decision-making process remains unclear.

The field of neonatology has some special features compared to other fields. For example, it must be considered that in a NICU, parents not only belong to the group of those (in-)directly affected, but also have to make decisions on behalf of their newborns. Decisions that are significantly influenced by the type of communication and at the same time affect the psychological well-being of the decision-makers.[8, 9, 17]

The evidence regarding the type of communication of unfavorable prognoses in the field of neonatology is still insufficient compared to other specialties, and consequently of great scientific interest. The study presented here addresses the question of which type of communication of an unfavorable prognosis is preferred by parents of very immature preterm infants, e.g., in the context of a severe complication. It is time to deal with such an important question not only theoretically but also in practice and to find out by questioning the parents of patients in which way they would like to receive such a prognosis. If the parents' preference is taken into account at the moment of diagnosis and especially at the moment of prognosis transmission, this is an important basis for future discussions, decision-making and for the best possible processing of the "history of the disease" or parental adaptation to the reality as parents of a child born immature and thus in some respects "fragile" instead of mature and healthy, which deviates from their original ideas. If parents express dissatisfaction with their child's NICU stay, this is mostly due to the fact that their children are well cared for, but their own, i.e. parental needs - often in connection with doctor-parent communication - are not sufficiently addressed.[18]

Of scientific interest in this context is whether and how communication can be geared to parental needs and preferences in the future and how this can contribute to improving the quality of care in neonatology in the long term in the sense of parent-centered, personalized communication. Parents of former preterm infants can provide valuable support thanks to their own lived experience of preterm birth, which can even be a life transformation experience for parents. There is a multitude of possibilities to give a voice to "former parents".[19-21] Especially in research projects like this one or in the scientific literature, this can be a valuable resource.

It is relevant to know that medical personnel more often give optimistic than pessimistic advice when communicating unfavorable prognoses.[9, 10, 17] Strategies of optimistic framing of unfavorable prognoses have been researched in this context.[10, 13] In this context, it is important, among other things, to define parental optimism

conceptually and to examine it more closely. This is the basis for research on the influence of a more or less optimistic framing of an unfavorable prognosis, since it must be assumed that the degree of optimism of a prognosis transmission is evaluated differently depending on the basic parental constitution (rather optimistic vs. rather pessimistic) and influences parental decision(s).
Optimism in this context is thus defined as parental expectation of the achievement of a certain goal. For example, the parents' goal could be that their child survives at all or without future impairment. The present study aims to find out whether parents with an optimistic view of life compared to those with a more pessimistic basic attitude, depending on a certain way of conveying a limiting prognosis, evaluate it differently and decide differently in the course of time. Optimistic framing of bad news is often understood as an attempt not to generate a feeling of hopelessness in patients (parents).[22, 23] There is also the assumption that in this approach the principle of hope, which is important for patients (parents), plays a decisive role in critical situations. In this context, there is a need to differentiate between the principles of hope and the aforementioned optimism.

Hope is complex and multimodal.[24] It finds expression, for example, in (general and/or specific) parental wishes for their child. While distinguishing hope from optimism in this way, it is possible to be hopeful and not optimistic at the same time. Despite an unfavorable prognosis, it is not unrealistic for parents to hope for the survival of their own child, even if they do not expect, i.e. cannot be optimistic, that it will survive.[25] Accordingly, it is possible for the consulting physician to maintain parental hope by the manner in which a prognosis is conveyed, regardless of its severity.[25] The realistic presentation of a poor prognosis in all its severity does not conflict with the communication of hope.[23, 26, 27] Rather, honesty promotes a sense of hope because it removes uncertainty and allows parents to make a decision consistent with the child's situation.[28] In this context, the question now arises whether an initial or basal high personal level of hope is reflected in a higher level of hope for one's own child even in the event of a serious complication with an unfavorable prognosis. The same applies to the opposite case of a high degree of hopelessness.

Recognizing the interindividual differences that exist between parents with regard to certain personality traits may be an important resource in choosing a way of communicating a limiting prognosis that is tailored to the parents and should therefore be investigated further. In addition to personality traits such as dispositional optimism, pessimism, and dispositional hopefulness (lack of hope), other possible factors influencing parental preference for a more or less optimistic communication style should be included. Therefore, in the context of this research project, parental coping with difficult life events (resilience), parental uncertainty (in)tolerance, and stress or dispositional anxiety will also be considered. It is of interest whether and how these factors influence the perception of the way prognosis is communicated, the physician's assessment, and a possible treatment decision. The aim is to investigate whether, according to expectation, uncertainty-intolerant parents react differently to different ways of communicating uncertainty (prognoses) than uncertainty-tolerant ones. It will also be examined whether there is a connection between anxiety or resilience in the sense of psychological resistance and parental preference for a certain type of prognosis communication.

Data from specialties other than neonatology suggest that optimistic advice is initially preferred and experienced as positive, as well as having a positive influence on the assessment of empathy and trustworthiness of medical staff. In addition, this is thought to increase trust in medical staff.[29] Current studies show that physicians often give overly optimistic advice and that the discrepancy in the assessment of the extent to which a prognosis is limited between healthcare professionals and patients or parents increases with the degree of optimism.[17] It can be assumed that physicians as well as patients (and their parents) assess the extent of the limitation of a prognosis more realistically if the prognosis is not conveyed optimistically. Excessive optimism can thus also have an unfavorable influence on the limitation of a prognosis as perceived by physicians and patients (parents), whereas less optimistic advice brings about greater agreement between both parties (concordance).[17] In particular, when the outcome resulting from the poor prognosis occurs, less optimistic counseling seems to be more favorable with respect to the relationship between physicians and patients and disease management.[23, 29] On the other hand, less optimistic counseling may also be experienced as negative, causing stress and thus complicating communication between the parties involved.[23] Thus, the extremely important question arises as to what influence the different ways of communicating an unfavorable prognosis have on the assessment of, e.g., the competence of the counselors, on the distress level of the counselees, or on the extent of perceived preparation for approaching (treatment) decisions.

In neonatology, there is a lack of empirical data with regard to the way in which unfavorable prognoses are communicated.[9, 12] At the same time, this specialty has very special demands on medical staff and patient parents in their role as surrogate decision makers. For this reason, the results of existing studies from other specialties cannot fulfill the claim that their results can be unconditionally transferred to the field of neonatology. In the context of this research project, therefore, the already elaborated, so far unanswered questions are to be taken up and decisively investigated.

2. Objectives of the research project, presentation of the scientific hypothesis.

Reports and recommendations published not only by medical professionals but also by professionals and at the same time experiencers, i.e., persons with lived experience of preterm birth and at the same time professional background as health care workers in the NICU (so-called POST, Parents on the Other Side of Treatment)[18, 30, 31], published reports and recommendations can be a valuable resource for improving care in neonatology. They promote an increase in knowledge regarding the parental experience of preterm birth and the challenging time in the NICU, as well as regarding the management of preterm parents in general or physician-parent communication in the context of complex situations in particular.[18, 30-33] In addition, it is known that parents want to be asked how they want information about their child to be communicated.[31] However, what type of communication, or in particular what level of optimism when communicating an unfavorable prognosis, is desired by parents in the NICU is virtually unknown and requires further research. It is also of great scientific interest what kind of communication positively influences parental perception of, for example, the degree of restriction of a prognosis or selected characteristics of the medical staff. The assessment of the medical staff can in turn influence the relationship between the medical staff and substitute decision makers and is thus an important resource in jointly finding and making the most appropriate (treatment) decision for the child in the given situation.

The following specific questions will be answered as part of the COPE-Trial:

2.1 Main research question - primary hypothesis

1. Parents prefer pessimistic framing of an unfavorable prognosis.

2.2 Secondary research questions - secondary hypotheses

1. With pessimistic framing, parental (state) anxiety is greater than with optimistic framing.
2. With pessimistic framing, parental satisfaction with communication is greater than with optimistic framing.
3. With pessimistic framing, parents estimate the conveyed outcomes more realistically than with optimistic framing.
    a. With pessimistic framing, parents show less deviation in the recall from the communicated relative probabilities (probabilistic data) regarding survival of the preterm infant (general or without impairment).
    b. With pessimistic framing, parents report a higher severity of the child's expected impairment.
    c. With pessimistic framing, parents report a higher degree of being informed regarding the prognosis.
4. With pessimistic framing, parents are less optimistic about the patient's future than with optimistic framing.
    a. With pessimistic framing, parents are less optimistic that the patient will survive the cerebral hemorrhage.
    b. With pessimistic framing, parents are less optimistic that the patient will survive without (cognitive/physical) impairment.
5. With pessimistic framing, parents express less hopefulness about the patient's future.
6. With pessimistic framing, parents indicate a higher degree of preparation to make a treatment decision together with the attending physician.
7. With pessimistic framing, the physician makes a better general impression on parents.
8. With pessimistic framing, parents rate the physician as more professional.
9. With pessimistic framing, parents rate the physician as less compassionate.
10. Physicians prefer pessimistic framing to an optimistic framing.
11. Physicians estimate the severity of prognosis more objectively when prognosis is framed pessimistically than when prognosis is framed optimistically.

III General planning

1. Summary of the study procedures

Within the framework of a randomized-controlled study in a parallel group design, parents of former patients of the NICU of the University Medical Center of the Johannes Gutenberg University Mainz will be included as subjects in the study collective (for inclusion and exclusion criteria, see IV). In order to capture the perception of the parents analogous to that of the transmitters of prognoses in everyday clinical practice, (assistant) physicians will also be included in the study according to predefined criteria (see ibid.).

The present research project is generally divided into two phases.
In the first phase, study participants will be recruited through personal and electronic contact by or with the study team. Personal contact means the combination of postal and telephone initial contact with each parent. To ensure that parents are prepared for a call from a study staff member, they will receive study information by mail in advance (approximately 1-2 weeks in advance). In addition to a brief overview of the background, content and procedures of the study as well as information on data protection, this information also contains the contact details of the study team, so that they can ask questions in writing or contact the team electronically if they are interested in participating in the study. Finally, during the telephone call, the parents are given detailed verbal information about the study (background, objectives, methodological procedure, opportunities for participation, source of further information, contact details of the study team). There is room for open questions. In addition, the inclusion and exclusion criteria are discussed with the parents. As soon as one of the parents meets one of the exclusion criteria, participation in the study is discouraged or directly discontinued. The second parent of the child can decide for or against participation independently of the parent excluded from study participation.

During the acquisition process families or related parents of a preterm infant are contacted together. However, in general, the parents are not treated as an entity, but as two independent subjects. This means that fathers and mothers, or both parents of the same child, do not process the study together, but separately, in order to experience the study intervention independently. This way, on the one hand, the involvement of individual parents is also guaranteed, should one parent not be interested in participating, not be present/existent, or not be allowed to participate if predefined exclusion criteria are met. On the other hand, experience shows that parents, for example, react differently to interviews, evaluate them differently, and accordingly place different demands on these interviews. For our study, therefore, it is interesting to consider both parents separately.
Parents who are interested in participating in the study and have been informed about the study will receive the electronic invitation to participate in the COPE-Trial with parents after the initial personal contact. In addition to brief instructions regarding the practical implementation of the study (from opening the link to completing the answering of all questions), as well as the detailed versions of the study information, the data protection information, and the consent form (as a PDF in the attachment), the parents receive their personal link to the online study in this e-mail. Parents will receive their individual password separately. This is a multi-digit access code - a combination of numbers and letters. Parents who belong together receive a code that is the same in the prefix (initial character) and otherwise differs. This makes it possible to mark related parents in the data set for statistical analysis without later being able to draw conclusions about individuals. All contacted families thus receive two codes as passwords. If a parent does not participate in the study, one of the codes remains unused and consequently there is no assignment of the parents in the data set. The codes are so-called serial numbers, which fulfill several functions: they function as a password, guarantee the one-time processing of the study, i.e. prevent multiple completion of the questionnaire by one person, and allow the assignment of related parents in the data set.

By clicking on the link, the participants are taken to the password-protected survey on the SoSci Survey online platform. Each link already contains the information to which study group the respective participant is randomly assigned (see explanation of randomization on p.26). Consequently, related parents of the same preterm infant receive the same link in order to know that they are assigned to the same study group as a parent pair. The reason for this procedure is that on the one hand all parents should (be able to) participate in the study as individuals, on the other hand at the same time the assumed common response tendency (covariance) of the parents of the same preterm infant should be taken into account if both participate in the study. The password, however, is partially different for both parents, i.e. individual. The use of centrally assigned access data (link and individual password) for the password-protected survey serves to protect the test persons and their data.

After completion of the first and before the start of the second study phase, concealed randomization will take place (see VI point 3 on p. 26), i.e. the random allocation of participants to our two study groups, which will experience different interventions in the second study part. To ensure that matched parents receive the same allocation and at the same time that couples as well as single participating parents (mothers only or fathers only) are distributed in a balanced way to both study groups, randomization is performed as described in point 3 of the

statistical analysis (p. 26). Parents interested in participating in the study are therefore asked during the information session (in writing and by telephone) to indicate whether one or both parents wish to participate when providing electronic feedback to the study team. If couples register with the study team using a common e-mail address, note the wish for participation of both parents in this e-mail address. If the parents register independently, please refer to the other participating parent in your feedback to allow the study team to send the same link to both parents.

The actual survey of the parents takes place online in the second phase of the study. This should enable the study participants to conduct the survey flexibly in terms of time and space. The study will be conducted using the online tool SoSci Survey (soscisurvey.de) developed in Munich, Germany. SoSci Survey allows secure surveys in terms of data protection: the program is data protection compliant according to DSGVO and BDSG and the data transmission is SSL-encrypted throughout.

The online study is divided into two parts:
The first part begins with an introduction followed by the digital version of the consent form. After reading this, the study participants must electronically agree to participate in the study via an instrument specially designed by SoSci Survey. Only with the digital signing of the consent form is it possible to process the online study. In case of non-consent, the online study is automatically terminated. If consent is given, the first study phase (baseline questionnaire) automatically begins with the recording of important characteristics of the test subjects and their preterm infants (see p.22).

Only in the second part of the study does the covert randomized assignment of the subjects to different processing areas. This is due to the fact that the parallel group design of the study provides for the two study groups to watch two videos - one the intervention (Video A), the other the control (Video B) - in immediate succession. The two study groups differ in the order in which the two videos are shown, i.e., the intervention and control (see Fig. 1). Advantages of this design are that all study participants act as their own control by watching both videos, and the question of comparability of groups with respect to confounding variables does not arise.

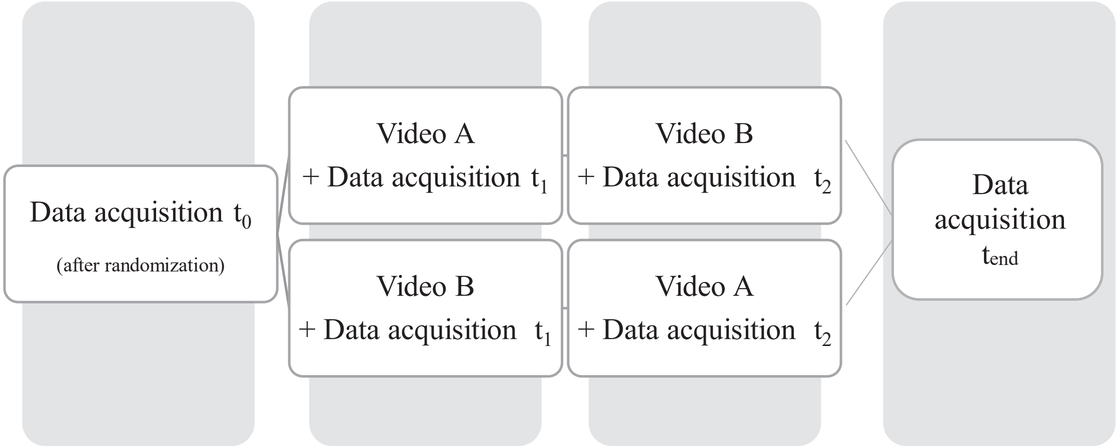

Fig. 1: Scheme of the second study phase.

The aim of the study is to simulate a consultation situation with transmission of an unfavorable prognosis for a very immature preterm infant. The unfavorable prognosis refers to one of the most common complications in neonatology and characteristic for very immature preterm infants - intraventricular hemorrhage with parenchymal involvement.

At the beginning of the second study section, the background is presented to the parents in an introductory video in the form of a short explanatory film (case vignette). The scenario of a premature baby with 23 5/7 weeks gestational age (GA) is described. The infant was born prematurely due to an amniotic infection syndrome. The infant subsequently experiences a complication of unilateral severe cerebral hemorrhage with parenchymal involvement on her third day of life. The participants also learn that the parents of the female preterm infant named Luisa are contacted by telephone by the attending physician for this reason. In the telephone conversation, the parents are asked to come to the ward in person for an important discussion.
Following the introductory video, the study participants watch the two videos one after the other, which correspond to the cinematic implementation of a simulated, i.e. fictitious, consultation situation with transmission of an unfavorable prognosis for a premature baby in different ways.

Video A (intervention): optimistic framing

Video B (control): pessimistic framing

Prognoses can only be predicted with great uncertainty in individual cases. For this reason, the transmission of prognostic data in both scenarios refers to outcome estimation data from scientific publications. [34, 35]

Scripted video vignettes were identified as suitable study material for the present study. Their conception, cinematic realization, and use were in accordance with the recommendations of Hillen and van Vliet.[36, 37] The development and use of video vignettes was guided by the specific research question, objectives, and particular requirements of this specific research project. The conception of the scripts was inspired by a study on the influence of more or less pessimistic communication on the perception of physician compassion from and in adult oncology.[29]

The case vignette as well as the two scripts for the two different scenarios were developed in an interdisciplinary way. The introductory video and the two videos (A and B) were shot with the support of a film team consisting of several actresses, a cameraman and camera assistance. In principle, the selected scenarios did not differ in content, but in the way the limiting prognosis was delivered (optimistic vs. pessimistic framing). The number of empathetic utterances is comparable, the different degree of optimism is achieved by variation of selected text passages. As a final thought, both scenarios contain a statement by the physician in the sense of reassurance of non-abandonment. In order to make it easier for the study participants to put themselves in the position of the parents in this consultation situation (empathization), the parents are seen from the front in the video. In the present case, a female physician communicates the prognosis. We chose a female doctor for reasons of uniformity of the videos as well as authenticity. It corresponds to the reality of most NICUs, including at the University Medical Center Mainz, that mainly women work as physicians in neonatology in this specialty.

The design of the study provides for parents included in the research project, after initial randomization, to first complete a baseline questionnaire (Data acquisition t_0 , see p.22) as a later basis for possible subgroup analyses. Following each of the two consecutive videos - regardless of the order in which they are presented - the subjects answer questions in the form of a post-intervention questionnaire (see Data acquisition $t_1 + t_2 + t_{end}$, p. 23ff).

2. Endpoints of the study

Primary endpoint

1. Parental preference for pessimistic versus optimistic message framing:
    a. Patient parents' preference for video B or A (dichotomous question with 1 = first video and 2 = second video).
    b. Rating of the desired level of optimism when an unfavorable prognosis is conveyed (unimodal, fully verbalized 7-point rating scale ranging from 1 = not at all optimistic to 7 = very optimistic).

Secondary endpoints

1. State anxiety is assessed using the German short version (5 items) of the State-Trait Anxiety Inventory (STAI-SKD) scale.[38] is used to assess state anxiety. Here, the change in parental (state) anxiety by/after viewing the first video is determined as the difference in state anxiety at time points t_0 and $t_{1.}$

2. Parental satisfaction is assessed using a fully verbalized 7-point rating scale (unimodal with 1 = not at all satisfied to 7 = very satisfied).

3. The realism of the parental assessment of the prognosis is determined by the degree of agreement between subjective assessment and objective ability to reproduce the transmitted prognostic data:
    a. Subjective assessment of the severity of the prognosis is based on a statement to be rated by the parents using a fully verbalized 7-point rating scale (1 = very bad to 7 = not bad at all).
    b. The objective ability to reproduce the transmitted information is based on the recall of the outcome data in numerical values (relative probabilities in %).
    c. The degree of parental preparedness for timely treatment decision-making is assessed using a fully verbalized 7-point rating scale (1 = not at all prepared to 7 = completely prepared).

Note: At the end of the study, parents are also asked to provide a general assessment regarding the importance of physician-parent interviews as well as the transmission of prognoses (i.e., probabilistic data) in particular (see Data acquisition t_{end} on p. 24).

4. The degree of parental optimism with regard to the child's future is determined by evaluating two statements (a) on survival of the complication per se and (b) on survival without impairment via the

respective fully verbalized 7-point rating scale (unimodal with 1= not optimistic at all to 7 = very optimistic). It is taken into account in the evaluation that the degree of optimism may depend on the participants' basic attitude towards life. The basic attitude towards life is therefore initially measured using the scale Optimism-Pessimism-2 (SOP-2).[39-41] recorded.

5. The degree of perceived hopefulness is assessed using a fully verbalized 7-point rating scale (unimodal with 1= not at all hopeful to 7 = very hopeful). A possible correlation of the individual expression of parental hope recorded in the baseline using the German version of the Herth-Hope Index (HHI-D)[42, 43] with the assessment of parental hope (lack of hope) following the video.

6. Parents rate on a fully verbalized 7-point rating scale how prepared they felt by the interview to make a treatment decision with the clinician in a timely manner (1 = not at all prepared and 7 = completely prepared).

7. The general impression of the consulting physician on the parents is determined using a fully verbalized 5-point rating scale (German school grades 1 to 5)

8. To assess physician professionalism, the German translation of a subscale of the Professional Performance Questionnaire adapted from [44] (verbal anchoring with German school grades 1 to 5; sum score: range 9 - 45 points) is used. A back-translation by a native speaker was performed.

9. Physician compassion was assessed with the help of the German-translated version of the Physician Compassion Questionnaire according to [45] (5 items, polarity profiles with 10 scale points each; sum score: range 5-50). A back-translation by a native speaker was performed.

Additionally for physicians:

10. In this case, the evaluation of a colleague questionnaire is used to assess the *colleague* advising in the video. [44].
11. A fully verbalized rating scale (range 1-7) is used to self-assess the individual approach of the participating physician.

IV. Study participants

1. **Complete inclusion criteria**

- Parents of former premature infants (birth weight < 1500 g and born after 31.12.2009)
- Postnatal treatment of a premature infant at University Medical Center of the Johannes-Gutenberg University Mainz
- Physicians with at least 6 months of work experience in a NICU.

Explanation: There will be two separate sub-projects. One study with parents as participants and one with physicians.

The study is to be carried out with the inclusion of the selected parents as well as medical personnel who are confronted with such complex scenarios in their daily work. It must be assumed that the special circumstances of the experience of a situation comparable to the scenario by parents in the past plays an important role in the influence of more or less optimistic framing of an unfavorable prognosis, for example, on the assessment of medical personnel as well as the preference of a certain type of prognosis transmission to be investigated. The expertise of the selected parental study collective is thus a prerequisite for gaining knowledge in the context of this survey. This study deliberately refrains from including parents who are actually acutely confronted with the situation of a serious complication of their preterm infant.

2. **Complete exclusion criteria**

- Lack of informed consent
- Insufficient knowledge of German language
- Self-reported acute, severe mental illness

3. **Are subjects included who have doubts about their ability to consent or who do not have the ability to consent?**

   No

4. **Will subjects lacking capacity to consent be included in the research project?**

   No.

5. **How are subjects recruited and is recruitment material used?**
   Patient parents will be recruited in person, i.e. by mail, telephone, and electronically or in the course of a medical consultation, e.g. at discharge or in the course of the developmental neurological follow-up for former preterm infants at Mainz University Medical Center, as well as by contacting and via the parents' association of preterm infants "Frühstart e.V.". If parents are interested in participating, they successively contact the study team in person or electronically. (Assistant) Physicians with at least 6 months of work experience will be recruited in person and electronically.

V. Study procedures

1. **Approach to participants**
   The recruitment of suitable study participants is carried out by the initially listed study personnel. The associated verification of compliance with the predefined inclusion and exclusion criteria serves to ensure the recruitment of suitable parents for the study.
   Following the acquisition (by post and personal telephone conversation), the information sheet, the consent form and the data protection information on the study are handed out to the test subjects electronically (e-mail). Accordingly, after receipt of the e-mail and before the start of the study, the parents can take sufficient time (at least 24 hours) to consent to or decline participation in the study. The second phase of the research project and thus participation in the study does not begin until the consent form is signed electronically. Participation can be withdrawn verbally or in writing before or at the beginning and at any time without giving reasons. Non-participation or early withdrawal from the study will not result in any disadvantages for the subjects. The same procedure for inclusion in the research project applies to female physicians.

2. **Study process and implementation (see Fig. 2).**
   The study is scheduled to begin on 04/01/2021 and is planned to last for one year, but at least until the minimum number of cases (n) is reached.
   After recruitment, randomization and inclusion in the study collective with electronic signing of the consent form, the study participants first complete the baseline questionnaire. After accessing the link and entering their individual password, the study participants can easily complete the study step by step from an Internet-enabled computer in the SoSci Survey online portal. When the number of cases reaches 2n, the study is terminated prematurely.

	Registration	Data acquisition	Intervention	Data acquisition	Intervention	Data acquisition	End
Time	t_{-1}	t_0	t_1	t_1	t_2	t_2	t_{end}
			Video		Video		
Registration							
Eligibility	x						
Consent	x	x					
Group assignment	x						
Intervention							
Video			A		B		
Video			B		A		
Evaluation							
Demographics		x					
Primary endpoint						x	
Secondary endpoints				x		x	x

Fig. 2: Study procedure and organization of data collection

The first part of the study with a short introduction, consent to participate in the study and answering the questions of the baseline questionnaire takes about 15 to 20 minutes. The time required for the second part of the study (main part with intervention) is also around 20 minutes for the test subjects (see Fig. 3).

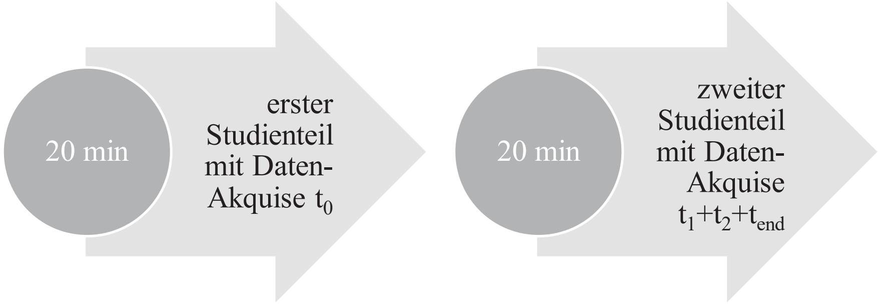

Fig. 3: respective time required for the two study sections

In the main part of the study, the test subjects first watch a short explanatory film before being shown the two videos one after the other in a different order, depending on their study group (see Fig. 1). After watching the first video, they are asked to comment on it by answering identical questions in each group. The questions following the two videos viewed one after the other are also identical in the two groups. After all questions have been answered, the study is considered complete. Upon completion of the survey, parents are thanked for their participation in the study and asked to close the browser window.

The following data on the study process will be collected:
Number of parents approached, number of parents excluded (including reasons: declined to participate, exclusion criteria, other reasons), number of parents included and randomized to the respective scenarios, number of parents discontinuing the study, number of parents finally analyzed (including reasons for non-analysis). The same applies to female physicians.

Data acquisition t_0 (baseline questionnaire)
As a basis of ensuring comparability and enabling subgroup analyses with regard to the primary and secondary endpoints of the study, sociodemographic data of the parents such as age, sex, sociocultural background (including language and religiosity/belief), highest educational attainment, employment, presence of medical expertise/knowledge, or medical background will be collected at baseline.

Since we assume that premature birth is a special life event for parents, they are asked how much they agree with the statement that the early birth of the child has changed their life, the family cohesion as well as the financial security of the family. To inquire about the influence of the premature birth on the family's instrumental resources, four items adapted from the "financial stress" subscale (F1-F4) of the Family Stress Questionnaire (FaBel questionnaire) were[46] formulated. Also, when asking about changes in family cohesion, an item of the subscale "coping problems" (F12) of the same questionnaire is used. This is followed by questions about the family (marital status, number of children in the household) and information about the premature baby (date of birth, gestational age of the premature baby at birth, birth weight, multiple birth status, fertility treatment yes/no, severe cerebral hemorrhage yes/no).

In addition, with regard to the evaluation of outcome data after study inclusion**,** the following specific (personality) characteristics of parents and medical staff will be recorded:

a. German version of the Brief Resilience Scale (BRS)[47-49]
b. The Optimism-Pessimism-2 Scale (SOP2)[39-41]
c. Herth Hope Index - German Version (HHI-D)[42, 43]
d. Uncertainty Tolerance Scale (UGTS)[50]
e. Patient Health Questionnaire (PHQ 4)[51, 52]
f. State-Trait Anxiety Inventory (STAI) Trate Scale.[53, 54]
g. German short version of the State-Trait Anxiety Inventory Scale (STAI-SKD)[38]

Data acquisition t_1 after the first video (A or B)

Parents are asked to rate the degree of optimism of prognosis communication on a 7 point rating scale (1 = not at all optimistic to 7 = very optimistic).

- Parents' (state) anxiety is assessed using the German short version (5 items) of the State-Trait Anxiety Inventory (STAI-SKD) scale.[38].
- Parental satisfaction with the way the clinician communicated the prognosis is assessed using a fully verbalized rating scale (range 1-7).
- The realism of parental assessment of prognosis is determined by the correlation between subjective assessment of the severity of prognosis (fully verbalized rating scale, range 1-7) and objective ability to reproduce the prognostic data conveyed. It will solicit a selection from percentages ranging from 0 to 100 percent in increments of ten (survival) or twenty-five (impairment).
- Parental level of optimism about the child's future (overall survival and survival without impairment) is assessed using a fully verbalized rating scale (range 1-7).
- A fully verbalized rating scale (range 1-7) is used to assess the degree of perceived hope (hopelessness).
- Using a fully verbalized rating scale (range 1-7), parents are asked whether they felt prepared to make a joint (treatment) decision with the physician in the announced follow-up interview.
- The general impression of the consulting physician on the parents is visualized by a fully verbalized rating scale (school grades 1 to 5, equal to the scale of the Professional Performance Questionnaire adapted to [44], sum score: 5-50) visualized.
- Physician professionalism is assessed using the German translation of a subscale of the Professional Performance Questionnaire (school grades 1 to 5, sum score: range 9 - 45 points) adapted from [44] determined.
- Physician compassion is assessed using the German translation of the Physician Compassion Questionnaire[45] in a slightly modified version adapted from [29]in a slightly modified version. The labels of the poles of the 5 items depicting the five dimensions of physician compassion were translated literally and back-translated by a native speaker, the scaling corresponds to a numerical rating scale with range 1-10.

For physicians additionally:

- In this case, the questionnaire *evaluation of a colleague is* used to evaluate the colleague according to [44].
- A fully verbalized rating scale (range 1-7) is used to self-assess the individual approach of the participating female physicians.

Data acquisition t_2 after the second video (B or A)

- Parents are asked to rate the degree of optimism of prognosis communication on a 7-point rating scale (1 = not at all optimistic to 7 = very optimistic).
- Preference for video A or B (primary endpoint) is captured using a dichotomous question (1 = first video and 2 = second video).

Data acquisition t_{end} on the completion of the study

- Participants are explicitly asked about their preference for (rather) pessimistic or (rather) optimistic communication of forecasts (fully verbalized 7-point rating scale with 1 = not at all optimistic to 7 = very optimistic).
- Parents are asked to provide an assessment regarding the importance of doctor-parent discussions in general and certain discussion content (transmission of prognostic data) in particular (4 items each and a fully verbalized 5-point rating scale with 1 = strongly disagree to 5 = strongly agree).
- Parents indicate whether they feel burdened by study participation in general or by watching the videos in particular (2 items and a 5-point rating scale with 1 = not at all to 7 = very much).

- Parents are informed that the study team is planning further research projects involving parents of former preterm infants in the future. Parents are invited to express their interest in participating in future studies by contacting the study team directly by e-mail.
- Physicians have the opportunity to comment on difficulties in communicating prognoses in everyday clinical practice. A free text field is available for this purpose.

3. **Rules for discontinuing the research project for the individual, as well as for the entire study**
   Only very low study-related risks are assumed for the test subjects (minimal risk). Individual subjects may discontinue the study at any time, e.g. if dealing with the subject matter is too emotionally stressful. In this case, a psychological consultation will be offered. If more than 50% of the subjects drop out of the study, the entire study will be terminated. This evaluation is carried out for every 20 subjects included.

VI. Statistics

1. Sample size calculation

Simplifying, the resulting number of cases is used as the number of parent pairs to be recruited, where one or both parents may consent. Tested the null hypothesis of balanced preference, period-corrected, with a chi quadrat test for independence in the four-field table defined by sequence and 'preference for video B vs. video A'. To detect an effect corresponding to the 3:2 preference ratio with a power of 80%, in the absence of a period effect, 194 families with evaluable data from at least one parent are needed. Assuming a significant period effect, the same treatment effect in sequence AB could result in a preference for video 1 with a probability of 0.50, whereas in sequence BA it would be 0.3077. The required number of cases would then be 204. Assuming that only one parent participates per family and that the dropout rate is 5%, 215 families would have to be recruited. At least 153 families would have to be recruited if both partners always participate and the agreement in the assessment corresponds to a kappa of 0.5. After inclusion of 50 to 70 families, a reassessment of the number of cases will be scheduled.

2. Statistical evaluation methods

The analysis of the primary endpoint 'preference B over A' is done by fitting a marginal logistic model with the endpoint 'second video was preferred' using the method of generalized estimating equations. For this purpose, parents are considered as clusters of scope 1 or 2. The exponentiated halved coefficient of the factor 'sequence' (A then B coded as 0, B then A coded as 1) is used as the period-corrected estimate of the odds for preference of intervention A. The test of the null hypothesis is performed as a Wald chi quadrat test at the two-tailed level 0.05.

3. Description of the randomization method

The concealment of allocation is performed in blocks of variable length, stratified into the three strata 'participation of mother only', 'participation of father only', 'participation of both parents'. Allocation will be done after recruitment of the parents by an employee of the University Medical Center of the Johannes Gutenberg University Mainz not involved in the acquisition outside the online acquisition software and will be done in the order of consent. Both parents receive the same allocation in each case.

VII Ethical and legal aspects

1. The study is conducted in accordance with currently valid national and international ethical and legal standards. The Declaration of Helsinki in its latest German version will be observed. The study protocol and substantial amendments will be registered with clinicaltrials.gov and the German Registry of Clinical Trials (DRKS) before the start of the study. The legal basis for the protection of personal data is the European Data Protection Regulation (DS-GVO). Personal data will only be processed after an effective declaration of consent (Art. 6 para. 1 letter c) DSGVO).
   The patient data are first collected digitally on the questionnaire. Subsequently, a code is assigned to each patient. The data is stored digitally by means of the patient code. The collection and storage of study-related data is thus exclusively in pseudonymized form.

2. **Information on the purpose and potential benefits or risks of the study.**
   The research project serves a purely scientific objective. In the long term, the study results should help to increasingly take into account individual parental needs and preferences with regard to physician-parent communication in neonatology and to strengthen parent-centered care in this special and particularly challenging specialty.
   Some parents of preterm infants describe their child's premature birth as a life-changing experience that requires them to reinvent themselves as parents and rewrite their own story.[30] Physicians should and can support parents in this process: through medical expertise and through successful communication. It is likely that the way medical information is communicated can influence parents' lived experience. The findings of this study should help to orient future communication in the context of uncertainty more towards the individual needs and preferences of parents as representatives of their children and direct experiencers and thus contribute to improving the quality of care in neonatology.
   Parents of former preterm infants can help in this process thanks to their own lived experience of preterm birth and the accompanying life transformation. This research project wants to draw attention to the importance of communication in the context of uncertainty on the one hand, and to the involvement of "former parents" in research projects on the other hand. It would be desirable in the near future to involve parents even more in neonatology or pediatric research in general and to do research with them rather than just about them. There are already concepts and scientific findings for the active involvement of "resource veteran parents" not only in research, but also in clinical care or teaching.[19-21]

   a. What are the potential benefits to the subjects of participating in the trial?

   Participating parents and physicians are unlikely to derive any direct individual benefits from the study. A possible group benefit may arise from improved counseling practices for future parents in a similar situation.

   b. What are the burdens on the parents?

   Watching the scenarios in video format and subsequently answering related questions may elicit an emotional response from parents. However, the risk of relevant exposure is estimated to be negligible.

3. **Information on the handling of the test person/patient data**

   As part of the research project, data such as age or gender are asked to describe the participants. In addition, they are asked to provide characteristic health data of their child, e.g. gestational age. All data collected are used to better assess and evaluate preferences with regard to the type of counseling in the context of transmitting a limiting prognosis. All information and statements made by the test persons in the course of the study will be treated with absolute confidentiality.

   This means that all study employees comply with the provisions of data protection law. Personal data are processed only after effective declaration of consent (Art. 6 para. 1 letter c) DSGVO). All data collected are stored on data carriers, processed exclusively on access-protected computers, and then stored safely and securely for ten years at the Center for Pediatrics and Adolescent Medicine at Mainz University Medical Center.
   To ensure that the personal data and the children's health data cannot be traced back to the subjects or their child, a code is generated (pseudonymization). The pseudonymization code is stored at an independent employee of the University Medical Center Mainz, i.e. not directly involved in the study.

Access to and evaluation of the data collected will be performed exclusively by the study director and staff directly involved in the study. The data will not be passed on to third parties. The publication of the study results takes place exclusively in anonymized form.

Participants have the right to obtain information about their personal data at any time (including the provision of a copy free of charge) and to request restriction, transfer, correction or deletion of this data. Furthermore, they may object to the processing of their data at any time (Art. 13-21 DS-GVO).

Responsible for data processing is Dr. med. André Kidszun. The responsible data protection officer is the State Data Protection Commissioner RLP (contact: poststelle@datenschutz.rlp.de).

4. **Insurance coverage is not necessary.**

VIII. References

1. Bastek TK, Richardson DK, Zupancic JA, Burns JP. Prenatal consultation practices at the border of viability: a regional survey. Pediatrics. 2005;116(2):407-13.
2. Haward MF, Gaucher N, Payot A, Robson K, Janvier A. Personalized Decision Making: Practical Recommendations for Antenatal Counseling for Fragile Neonates. Clin Perinatol. 2017;44(2):429-45.
3. Trujillo JA, Fernandez Y, Ghafoori L, Lok K, Valencia A. Interdisciplinary Family Conferences to Improve Patient Experience in the Neonatal Intensive Care Unit. Health & social work. 2017;42(4):241-6.
4. Partridge JC, Martinez AM, Nishida H, Boo NY, Tan KW, Yeung CY, et al. International comparison of care for very low birth weight infants: parents' perceptions of counseling and decision-making. Pediatrics. 2005;116(2):e263-71.
5. Lantos JD. Ethical Problems in Decision Making in the Neonatal ICU. The New England journal of medicine. 2018;379(19):1851-60.
6. Walter JK, Ross LF. Relational autonomy: moving beyond the limits of isolated individualism. Pediatrics. 2014;133 Suppl 1:S16-23.
7. Fangerau H. Ethik - eine Einführung. In: T Noak HF, J Vögele, editor. Geschichte, Theorie und Ethik der Medizin. 1 ed. München: Urban & Fischer; 2007. p. 1-6.
8. Glare PA, Sinclair CT. Palliative medicine review: prognostication. Journal of palliative medicine. 2008;11(1):84-103.
9. Boss RD, Lemmon ME, Arnold RM, Donohue PK. Communicating prognosis with parents of critically ill infants: direct observation of clinician behaviors. Journal of perinatology : official journal of the California Perinatal Association. 2017;37(11):1224-9.
10. Leydon GM. 'Yours is potentially serious but most of these are cured': optimistic communication in UK outpatient oncology consultations. Psycho-oncology. 2008;17(11):1081-8.
11. Enzinger AC, Zhang B, Schrag D, Prigerson HG. Outcomes of Prognostic Disclosure: Associations With Prognostic Understanding, Distress, and Relationship With Physician Among Patients With Advanced Cancer. Journal of clinical oncology : official journal of the American Society of Clinical Oncology. 2015;33(32):3809-16.
12. Boss RD, Donohue PK, Larson SM, Arnold RM, Roter DL. Family Conferences in the Neonatal ICU: Observation of Communication Dynamics and Contributions. Pediatric critical care medicine : a journal of the Society of Critical Care Medicine and the World Federation of Pediatric Intensive and Critical Care Societies. 2016;17(3):223-30.
13. Graugaard PK, Rogg L, Eide H, Uhlig T, Loge JH. Ways of providing the patient with a prognosis: a terminology of employed strategies based on qualitative data. Patient Educ Couns. 2011;83(1):80-6.
14. Harvey ME, Redshaw ME. Qualitative study of the clinician-parent interface in discussing prognosis following MRI and US imaging of preterm infants in the UK. BMJ open. 2016;6(9):e011472.
15. van Vliet LM, van der Wall E, Plum NM, Bensing JM. Explicit prognostic information and reassurance about nonabandonment when entering palliative breast cancer care: findings from a scripted video-vignette study. Journal of clinical oncology : official journal of the American Society of Clinical Oncology. 2013;31(26):3242-9.
16. Anderson WG, Cimino JW, Ernecoff NC, Ungar A, Shotsberger KJ, Pollice LA, et al. A multicenter study of key stakeholders' perspectives on communicating with surrogates about prognosis in intensive care units. Annals of the American Thoracic Society. 2015;12(2):142-52.
17. Robinson TM, Alexander SC, Hays M, Jeffreys AS, Olsen MK, Rodriguez KL, et al. Patient-oncologist communication in advanced cancer: predictors of patient perception of prognosis. Supportive care in cancer : official journal of the Multinational Association of Supportive Care in Cancer. 2008;16(9):1049-57.
18. Janvier A, Lantos J. Ethics and etiquette in neonatal intensive care. JAMA Pediatr. 2014;168(9):857-8.
19. Bourque CJ, Dahan S, Mantha G, Robson K, Reichherzer M, Janvier A. Improving neonatal care with the help of veteran resource parents: An overview of current practices. Semin Fetal Neonatal Med. 2018;23(1):44-51.
20. Dahan S, Bourque CJ, Reichherzer M, Ahmed M, Josée P, Mantha G, et al. Beyond a Seat at the Table: The Added Value of Family Stakeholders to Improve Care, Research, and Education in Neonatology. The Journal of pediatrics. 2019;207:123-9.e2.
21. Janvier A, Bourque CJ, Dahan S, Robson K, Barrington KJ. Integrating Parents in Neonatal and Pediatric Research. Neonatology. 2019;115(4):283-91.
22. Christakis NA, Iwashyna TJ. Attitude and self-reported practice regarding prognostication in a national sample of internists. Archives of internal medicine. 1998;158(21):2389-95.
23. Nyborn JA, Olcese M, Nickerson T, Mack JW. "Don't Try to Cover the Sky with Your Hands": Parents' Experiences with Prognosis Communication About Their Children with Advanced Cancer. Journal of palliative medicine. 2016;19(6):626-31.
24. Feudtner C. The breadth of hopes. The New England journal of medicine. 2009;361(24):2306-7.

25. Sisk BA, Malone JR. Hope, Optimism, and Compassionate Communication. JAMA Pediatr. 2018;172(4):311-2.
26. Winner M, Wilson A, Ronnekleiv-Kelly S, Smith TJ, Pawlik TM. A Singular Hope: How the Discussion Around Cancer Surgery Sometimes Fails. Annals of surgical oncology. 2017;24(1):31-7.
27. Mack JW, Wolfe J, Cook EF, Grier HE, Cleary PD, Weeks JC. Hope and prognostic disclosure. Journal of clinical oncology : official journal of the American Society of Clinical Oncology. 2007;25(35):5636-42.
28. Hagerty RG, Butow PN, Ellis PM, Lobb EA, Pendlebury SC, Leighl N, et al. Communicating with realism and hope: incurable cancer patients' views on the disclosure of prognosis. Journal of clinical oncology : official journal of the American Society of Clinical Oncology. 2005;23(6):1278-88.
29. Tanco K, Rhondali W, Perez-Cruz P, Tanzi S, Chisholm GB, Baile W, et al. Patient Perception of Physician Compassion After a More Optimistic vs a Less Optimistic Message: A Randomized Clinical Trial. JAMA oncology. 2015;1(2):176-83.
30. Janvier A, Lantos J, Aschner J, Barrington K, Batton B, Batton D, et al. Stronger and More Vulnerable: A Balanced View of the Impacts of the NICU Experience on Parents. Pediatrics. 2016;138(3).
31. Haward MF, Lantos J, Janvier A. Helping Parents Cope in the NICU. Pediatrics. 2020;145(6).
32. Janvier A, Barrington K, Farlow B. Communication with parents concerning withholding or withdrawing of life-sustaining interventions in neonatology. Semin Perinatol. 2014;38(1):38-46.
33. Lizotte MH, Barrington KJ, Sultan S, Pennaforte T, Moussa A, Lachance C, et al. Techniques to Communicate Better With Parents During End-of-Life Scenarios in Neonatology. Pediatrics. 2020;145(2).
34. Merhar SL, Tabangin ME, Meinzen-Derr J, Schibler KR. Grade and laterality of intraventricular haemorrhage to predict 18-22 month neurodevelopmental outcomes in extremely low birthweight infants. Acta paediatrica (Oslo, Norway : 1992). 2012;101(4):414-8.
35. De Vries LS, Van Haastert IL, Rademaker KJ, Koopman C, Groenendaal F. Ultrasound abnormalities preceding cerebral palsy in high-risk preterm infants. The Journal of pediatrics. 2004;144(6):815-20.
36. Hillen MA, van Vliet LM, de Haes HC, Smets EM. Developing and administering scripted video vignettes for experimental research of patient-provider communication. Patient Educ Couns. 2013;91(3):295-309.
37. van Vliet LM, Hillen MA, van der Wall E, Plum N, Bensing JM. How to create and administer scripted video-vignettes in an experimental study on disclosure of a palliative breast cancer diagnosis. Patient Educ Couns. 2013;91(1):56-64.
38. Englert C, Bertrams A, Dickhäuser O. Entwicklung der Fünf-Item-Kurzskala STAI-SKD zur Messung von Zustandsangst. Zeitschrift für Gesundheitspsychologie. 2011;19(4):173-80.
39. Kemper CJ BC, Kovaleva A, Rammsted B. Eine Kurzskala zur Messung von Optimismus-Pessimismus - Die Skala Optimismus-Pessimismus-2 (SOP2). GESIS: Köln. 2012;GESIS Working Papers(2012|15).
40. Kemper CJ, Beierlein C, Kovaleva A, Rammstedt B. Entwicklung und Validierung einer ultrakurzen Operationalisierung des Konstrukts Optimismus-Pessimismus. Diagnostica. 2013;59(3):119-29.
41. Kemper CJ, Wassermann M, Hoppe A, Beierlein C, Rammstedt B. Measuring Dispositional Optimism in Large-Scale 					Studies. European Journal of Psychological Assessment. 2017;33(6):403-8.
42. Herth K. Abbreviated instrument to measure hope: development and psychometric evaluation. J Adv Nurs. 1992;17(10):1251-9.
43. Geiser F, Zajackowski K, Conrad R, Imbierowicz K, Wegener I, Herth KA, et al. The German Version of the Herth Hope Index (HHI-D): Development and Psychometric Properties. Oncol Res Treat. 2015;38(7-8):356-60.
44. Campbell JL, Richards SH, Dickens A, Greco M, Narayanan A, Brearley S. Assessing the professional performance of UK doctors: an evaluation of the utility of the General Medical Council patient and colleague questionnaires. Quality & safety in health care. 2008;17(3):187-93.
45. Fogarty LA, Curbow BA, Wingard JR, McDonnell K, Somerfield MR. Can 40 seconds of compassion reduce patient anxiety? Journal of clinical oncology : official journal of the American Society of Clinical Oncology. 1999;17(1):371-9.
46. Ravens-Sieberer U, Morfeld M, Stein RE, Jessop DJ, Bullinger M, Thyen U. [The testing and validation of the German version of the impact on family scale in families with children with disabilities]. Psychother Psychosom Med Psychol. 2001;51(9-10):384-93.
47. Smith BW, Dalen J, Wiggins K, Tooley E, Christopher P, Bernard J. The brief resilience scale: assessing the ability to bounce back. International journal of behavioral medicine. 2008;15(3):194-200.
48. Kunzler AM, Chmitorz A, Bagusat C, Kaluza AJ, Hoffmann I, Schäfer M, et al. Construct Validity and Population-Based Norms of the German Brief Resilience Scale (BRS). Eur J Health Psychol. 2018;25(3):107-17.
49. Chmitorz A, Wenzel M, Stieglitz RD, Kunzler A, Bagusat C, Helmreich I, et al. Population-based validation of a German version of the Brief Resilience Scale. PLoS One. 2018;13(2):e0192761.
50. Dalbert C. UGTS. Ungewissheitstoleranzskala [Verfahrensdokumentation aus PSYNDEX Tests-Nr. 9003844, Autorenbeschreibung, Fragebogen Deutsch und Fragebogen Englisch]. In Leibniz-Zentrum für Psychologische Information und Dokumentation (ZPID) (Hrsg), Elektronisches Testarchiv Trier: ZPID https://doiorg/1023668/psycharchives328. 2002.

51. Kroenke K, Spitzer RL, Williams JB, Lowe B. An ultra-brief screening scale for anxiety and depression: the PHQ-4. Psychosomatics. 2009;50(6):613-21.
52. Löwe B, Wahl I, Rose M, Spitzer C, Glaesmer H, Wingenfeld K, et al. A 4-item measure of depression and anxiety: validation and standardization of the Patient Health Questionnaire-4 (PHQ-4) in the general population. J Affect Disord. 2010;122(1-2):86-95.
53. Spielberger CD GRLR. State-Trait Anxiety Inventory, Manual for the State-Trait Anxiety Inventory. Palo Alto, CA: Consulting Psychologist Press; 1970.
54. Laux L GP, Schaffner P & Spielberger CD. „Das State-Trait-Angstinventar (Testmappe mit Handanweisung, Fragebogen STAI-G Form X 1 und Fragebogen STAI-G Form X 2)". Weinheim: Beltz; 1981.

IX. Signatures

Mainz, 05.02.2021

....
André Kidszun, MD, MA; Principle Investigator

....
Dr. Jochem König, Biostatistician
