# Supplementary material for: Optimistic vs Pessimistic Message Framing in Communicating Prognosis to Parents of Very Preterm Infants: The COPE Randomized Clinical Trial
Source: JAMA Netw Open. 2024 Feb 23;7(2):e240105. doi: 10.1001/jamanetworkopen.2024.0105 (PMC10891472; doi:10.1001/jamanetworkopen.2024.0105)
Supplement: Supplement 3. — Data Sharing Statement [file jamanetwopen-e240105-s003.pdf]

Data Sharing Statement

Forth. Effects of Optimistic vs Pessimistic Message Framing in Communicating Prognosis to Parents of Very Preterm Infants. *JAMA Netw Open*. Published February 21, 2024. doi:10.1001/jamanetworkopen.2024.0105

Data
Data available: No

Additional Information
Explanation for why data not available: Participant-level data will not be disclosed to third parties for data protection reasons. The final trial dataset is accessed only by the principal investigator and research team members directly involved in the COPE-Trial. Study-related materials (see Data Sharing Statement) will either be part of the publication or will be available upon reasonable request.
